# Active Deformation Across the Western Anatolian Extensional Province (Türkiye) From Sentinel‐1 InSAR

**DOI:** 10.1029/2023TC008086

**Published:** 2024-11-24

**Authors:** Manuel Diercks, Ekbal Hussain, Zoë K. Mildon, Sarah J. Boulton, Milan Lazecký

**Affiliations:** ^1^ School of Geography Earth and Environmental Sciences University of Plymouth Plymouth UK; ^2^ British Geological Survey Natural Environment Research Council Environmental Science Centre Nottingham UK; ^3^ COMET School of Earth and Environment University of Leeds Leeds UK

**Keywords:** active tectonics, normal faulting, geodesy, decomposing InSAR velocities, Gediz Graben, uplift/subsidence

## Abstract

Quantifying interseismic deformation of fault networks which are predominantly deforming in a north‐south direction is challenging, because GNSS networks are usually not dense enough to resolve deformation at the level of individual faults. The alternative, interferometric synthetic aperture radar (InSAR), provides high spatial resolution but is limited by a low sensitivity to N‐S motion. We study the active normal fault network of Western Türkiye, which is undergoing rapid N‐S extension, using InSAR. Since most faults in the study region are normal faults, we overcome the low N‐S sensitivity by focusing on the vertical deformation component, which presents its own challenges. Sediment‐filled grabens show rapid anthropogenically induced subsidence, whereas urban areas tend toward erroneous uplift signals. Additionally, the morphological relief results in topographic and atmospheric disturbances of the InSAR signal. Our solution to these challenges is a systematic analysis of the high‐resolution vertical velocity field to deduce insights into regional deformation patterns, combined with detailed investigations of deformation along individual faults in the Western Anatolian Extensional Province. We show that tectonic deformation in the large graben systems is not restricted to the main faults. Smaller and seemingly less active faults are accommodating strain, favoring a continuum model of deformation over block models. We also observe a potential correlation between recent seismicity and active interseismic surface deformation. Observed deformation rates provide an estimate of current activity for many faults in the region. We discuss the potential and limitations of InSAR time series analysis for extensional regimes.

## Key Points

- Vertical interferometric synthetic aperture radar velocity field reveals shifts from uplift to subsidence spatially correlated with faults and fault splays
- Results indicate spatial variation in uplift rates along faults and could be used to infer the relative activity of faults or fault splays
- Geodetic deformation rates do not generally agree with (mostly exceed) long‐term (Holocene) deformation patterns

## 1 Introduction

**Figure 1:**
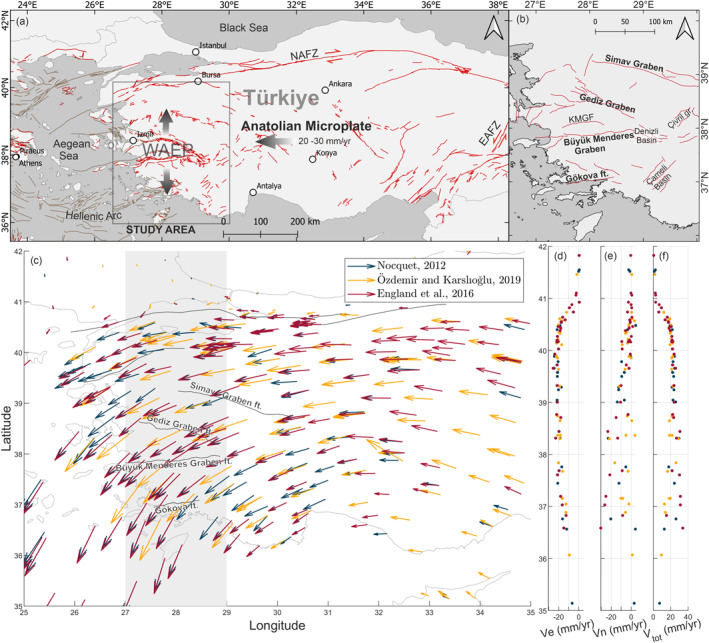
(a) Tectonic setting and major active fault zones of the Eastern Mediterranean (after Emre et al. (2018) and Ganas et al. (2013, 2023)). Box indicates the extent of the study area. NAFZ: North Anatolian Fault Zone; EAFZ: East Anatolian Fault Zone (b) Simplified fault network of the WAEP, Western Anatolian Extensional Province; KMGF, Küçük Menderes Graben Fault. (c) GNSS velocity field of Türkiye with respect to stable Eurasia. Gray‐shaded area marks the profile depicted in panels (d–f). GNSS velocities relative to stable Eurasia, hence the increase in both E‐W and N‐S components with distance to the NAFZ.

Fialko et al. (2001) and Wright et al. (2004) first described the process of decomposing interferometric synthetic aperture radar (InSAR) line‐of‐sight (LOS) signals into east‐west, north‐south and vertical components. Today, with the availability of high performance computing, InSAR time series, and improved satellite systems (namely the Sentinel‐1 system), this process has become a well‐established application. Here we study the Western Anatolian Extensional Province (WAEP), a region undergoing rapid N‐S orientated extension, using Sentinel‐1 InSAR time series. A difficulty of studying this region is the inherently poor InSAR sensitivity to movements in direction of the satellite's orbit (Wright et al., 2004). Since the Sentinel‐1 satellites (similar to previous SAR missions) are on approximately N‐S‐oriented orbits, the LOS velocity is significantly less sensitive to north‐south deformation compared to movements in the vertical and east‐west directions. GNSS studies (see Figure 1) show that Western Anatolia is undergoing rapid N‐S extension of ∼ 20 mm/yr (Aktug et al., 2009; McClusky et al., 2000) across a series of graben structures (McKenzie, 1972; Ten Veen et al., 2009) that have hosted large infrequent earthquakes $\leq M_{W}$ 7.0 (Eyidoğan & Jackson, 1985). The current state of activity on the graben fault systems is still not fully understood and so investigations into the regional fault network and deformation patterns can contribute to an understanding of fault activity and therefore seismic hazard.

The regional deformation is mostly accommodated by ∼ E‐W trending normal faults, mostly associated with the graben structures (Bozkurt & Sözbilir, 2004; Ten Veen et al., 2009). Since the extraction of reliable N‐S deformation is challenging, we focus our analysis of the active faults with vertical deformation rates. However, while InSAR is highly sensitive to vertical movements, this comes with a different challenge; the studied faults are mainly graben‐bounding faults, separating flat, sediment‐filled basins covered by agricultural land from mountainous areas. Consequently, the effects of topography, atmosphere and subsidence, owing to ground water extraction in the grabens, swamp the tectonic signal and complicate the ability to quantify or even detect tectonic subsidence (Aslan et al., 2022; Hastaoglu et al., 2023; Imamoglu et al., 2022). Therefore, the key challenge is to distinguish tectonic movements from other confounding influences. We navigate this problem by focusing on the footwall uplift of normal faults and neglecting the hangingwall deformation. While subsidence in the basin (hangingwall of normal faults) can have a variety of causes, footwall uplift can be mainly attributed to tectonic factors.

We quantify footwall uplift rates along active faults in the region and compare the spatial uplift patterns with the mapped fault traces. This provides insights into the activity of individual faults and fault splays, which are not detectable with other techniques.

## 2 Surface Deformation in the Western Anatolian Extensional Province

### 2.1 Regional Tectonics and Seismic Activity

Driven by the collision of the African, Eurasian and Arabian plates, the Anatolian microplate escapes westward between the North Anatolian (NAFZ) and East Anatolian (EAFZ) Fault Zones at a rate of 20–30 mm/year (Kurt et al., 2023). Owing to this movement, combined with roll back from the Hellenic Arc subduction zone, western Anatolia and parts of the Aegean Sea are undergoing N‐S extension at rates of ∼ 20 mm/year, forming the Western Anatolian Extension Province (Aktug et al., 2009; Jackson, 1994; McClusky et al., 2000; McKenzie, 1972, 1978; Taymaz et al., 2007). The dominant style of deformation in the WAEP is normal faulting on ∼ E‐W‐trending faults, forming a series of elongated basins (grabens). In the eastern Aegean Sea and coastal regions of Anatolia, often referred to as the “İzmir‐Balıkesir transfer zone,” a significant right‐lateral component of deformation is expressed in active strike‐slip deformation on ∼ NE‐SW‐trending faults (Uzel et al., 2012). This is also confirmed by strike‐slip earthquakes occurring in the region (e.g., Benetatos et al., 2006).

The most prominent structures in the study area are the E‐W‐trending Gediz and Büyük Menderes Graben, the Simav Graben in the north and the Gulf of Gökova at the Mediterranean coast (Figure 1). Other basins, predominantly bounded by active, NW‐SE or NE‐SW‐trending normal faults, are distributed across the WAEP. For our study we use a simplified fault network modified from the active fault database (Emre et al., 2018) and the accompanying active fault map series (1:250k scale). Faults in the Denizli Basin after Koçyiğit (2005), Çameli region after Alçiçek et al. (2006) and Yang et al. (2020). Fault traces were simplified to single lines and the location of the fault traces was modified based on morphology (DEM) and vertical deformation signals, where applicable.

### 2.2 Tectonic and Non‐Tectonic Surface Deformation

Hooper et al. (2012) and Weiss et al. (2020) computed the InSAR line‐of‐sight (LOS) velocity fields throughout Anatolia. Weiss et al. (2020) decomposed LOS velocities of entire Türkiye into east‐, north‐ and vertical components, though no detailed analysis and discussion of individual faults, deformation rates and regional uplift and subsidence patterns in Western Anatolia were done.

Surface deformation and aseismic creep is documented in several locations in the WAEP, for example, in the Afyon‐Akşehir Graben (Özkaymak et al., 2019). Particularly fast deformation rates are observed at the Sarigöl fault, the eastern segment of the Gediz Graben system, which ruptured in the 1969 $M_{W}$ 6.9 Alaşehir earthquake (Arpat & Bıngöl, 1969; Eyidoğan & Jackson, 1985). Vertical deformation at the fault was 70–87 mm/yr between July 2017 and 2020, inferred from precise leveling studies (Doğan et al., 2022). Other studies obtained vertical deformation of 60–85 mm/yr over a 10‐year period (Koca et al., 2011) or up to 90 mm/yr (Poyraz et al., 2019).

Most of the surface deformation observed is owing to subsidence in the grabens related to falling ground water levels, particularly in the summer. Since minor deformation continues throughout winter and spring, Doğan et al. (2022) conclude that tectonic creep also contributes to the observed vertical deformation, possibly in a range of ∼ 20 mm/yr. When removing the seasonal signal, which is mainly caused by groundwater level changes, from the time series, Hastaoglu et al. (2023) determined between 10 and 62 mm/yr of subsidence in the graben. Subsidence related to ground water level changes is known from multiple basins across the region (Aslan et al., 2022; Imamoglu et al., 2022). It generally exceeds tectonic deformation rates and is difficult to deconvolute from the tectonic subsidence. Agriculture and geothermal plants (Baba et al., 2015) are mostly restricted to the hangingwall of the active faults, therefore, we focus our analyses on the uplift signal of normal faults.

### 2.3 Seismicity During the Observation Period

**Figure 2:**
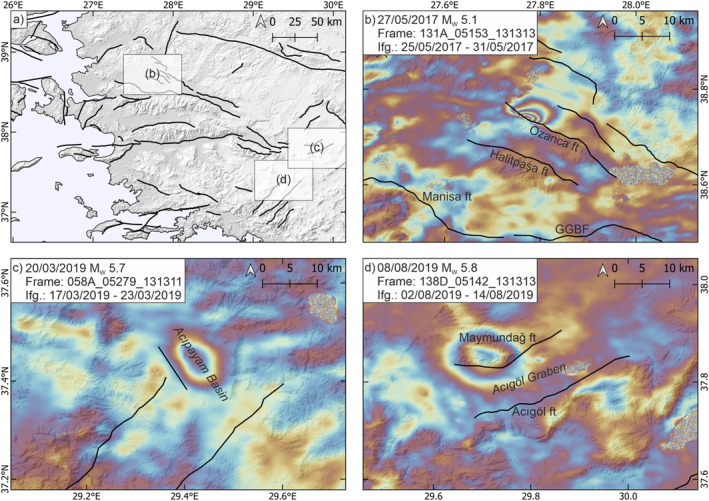
Interferograms of earthquakes of $M_{W}\;>$ 5.0 between 2014 and 2023 which caused observable surface displacements. (a) Overview map showing the locations of ruptures. (b) 27 May 2017 Ozanca Fault earthquake. (c) 20 March 2019 Acıpayam Basin earthquake. (d) 08 August 2019 Acıgöl Basin earthquake, likely rupturing the Acıgöl Fault. Frames and interferogram time spans are given in figure legends.

Eleven earthquakes of $M_{W}\;>$ 5.0 occurred in the study region within the observation period (Storchak et al., 2013), three of these caused surface displacement observable in single interferograms (Lazecký et al., 2020). On 27 May 2017 a $M_{W}$ 5.1 earthquake (Storchak et al., 2013) ruptured the Ozanca Fault, south of Akhisar in the western Gediz Graben (Figure 2b). On 20 March 2019, a $M_{W}$ 5.7 earthquake, which was also studied by Yang et al. (2020) and Nissen et al. (2022), occurred on an unknown blind fault in the Acıpayam Basin (Figure 2c). The source fault is referred to as the Acıpayam Basin Fault here. Another earthquake occurred below the northern margin of the Acıgöl Basin (Nissen et al., 2022) on 8 August 2019, with $M_{W}$ 5.8 and surface deformation observable in the descending Sentinel‐1 track (Figure 2d). An earthquake swarm of multiple shocks with $M_{W}∼$ 5.0 occurred north of Akhisar, around the Gelenbe Fault, in January and February 2020, with the largest recorded shock of $M_{W}$ 5.1 (Storchak et al., 2013). This was followed by a $M_{W}$ 5.3 earthquake in the same region, however no surface deformation is observable in interferograms (see Supplement 4 for interferograms of these events). Six more earthquakes of $M_{W}\;>$ 5.0 in the region are listed in the revised ISC catalog (Storchak et al., 2013), though none of these shows any surface displacement in interferograms provided by the LiCS portal (Lazecký et al., 2020).

## 3 Methods

### 3.1 Preparing InSAR and GNSS Velocities

We computed InSAR time series of six ascending and five descending frames (Table 1, Supplement 9) using LiCSBAS (Morishita et al., 2020) with data downloaded from the LiCSAR portal (Lazecký et al., 2020). This analysis included atmospheric corrections using GACOS data (Yu et al., 2018).

**Table 1:** Sentinel‐1 Frames, Time Span Covered, and Number of Interferograms (*n* ifgs) Used to Calculate Time Series

Frame ID	Geometry	Start date	End date	*n* ifgs
036D_04976	Descending	13/03/2015	30/01/2023	1,258
036D_05175	Descending	08/10/2014	28/06/2022	1,003
138D_04954	Descending	15/10/2014	29/07/2022	1,173
138D_05142	Descending	15/10/2014	13/01/2023	1,498
138D_05325	Descending	08/11/2014	29/07/2022	1,147
058A_04914	Ascending	09/01/2014	19/01/2023	1,358
058A_05086	Ascending	09/10/2014	27/08/2021	916
058A_05279	Ascending	09/01/2014	09/09/2022	1,293
131A_04951	Ascending	02/01/2018	31/12/2022	1,004
131A_05153	Ascending	07/11/2014	28/07/2022	1,053
131A_05336	Ascending	07/11/2014	31/12/2022	1,165

Following the approach of Hussain et al. (2016), the InSAR LOS velocities were referenced to a stable Eurasia reference frame, using three sets of GNSS velocity data (England et al., 2016; Nocquet, 2012; Özdemir & Karslıoğlu, 2019). We combine the data, averaging values for duplicate stations, and calculate the average InSAR LOS velocity in a square of ∼ 1 ${\text{km}}^{2}$ around each GNSS station. GNSS north $\left(V_{n}\right)$ and east $\left(V_{e}\right)$ velocities are converted into LOS velocities using the InSAR LOS vector components ($p_{x}$ and $p_{y}$) for the east and north directions, assuming that proportions of InSAR velocities are comparable to GNSS velocities: $LOS_{\mathrm{gnss}}=p_{x}\times V_{e}+p_{y}\times V_{n}$. This step was later repeated with newly released GNSS data from Kurt et al. (2023), with negligible differences in the resulting InSAR LOS velocities (average differences of 0.1090 mm/yr for ascending and 0.0262 mm/yr for descending LOS velocities, see Figure S3 in Supporting Information S1).

We then determine the best‐fit planes through the InSAR and GNSS LOS velocities. The difference between both planes reflects the difference of reference frames between the GNSS (relative to stable Eurasia) and the InSAR velocities, and is subsequently removed from the InSAR LOS velocity field. The procedure is repeated for each InSAR frame.

To use multiple InSAR data sets, combined with GNSS velocities, they must be on the same geographic grid. We therefore create a grid covering the study area from 26 to 31°E and 36 to 40.5°N, with a grid size of 0.0009° (∼ 100 m). We interpolate the GNSS velocities onto this grid, and then resample all InSAR LOS velocities on the grid, using the nearest‐neighbor method and preserving empty pixels.

The LOS velocities differ slightly between frames, even for frames on the same track. These differences result in artificial steps at frame boundaries in the combined velocity fields and later inversion results, and could mislead interpretations when falsely identified as natural features in the surface deformation rates. To reduce these artifacts, we apply another correction step, without changing the relative signals within each frame. For each geometry, one reference frame is picked (descending 138D 05142 and ascending 131A 05153), which is located in the center of the study area and shows reasonably good time series results. The secondary frame with the largest overlap area (omitting empty pixels) with the reference frame is determined. To adjust the velocity field to a similar range in velocities, the secondary frame $\left(LOS_{\sec}\right)$ is corrected by the standard deviation σ of the overlapping parts of the reference frame: $LOS_{\sec}^{\mathrm{adj}}=LOS_{\sec}\times \sigma_{\mathrm{ref}}/\sigma_{\sec}$. Then the median of velocities of both frames in the overlapping area is determined and the reference frame is corrected: $LOS_{\sec}^{\mathrm{adj}}=LOS_{\sec}+m_{\mathrm{ref}}-m_{\sec}$, where $m_{\mathrm{ref}},m_{\sec}$ is the median of the reference/secondary LOS velocities, respectively, in the overlapping area. This process is repeated for all frames, each time the reference area is enlarged by the newly referenced frame. Before the inversion, all frames are merged on the same track into single data sets (two ascending and two descending tracks), averaging overlapping pixels.

### 3.2 Inversion of the Vertical and East‐West Components

The LOS velocity can be decomposed into the three components of displacement, $D_{E}$, $D_{N}$, and $D_{U}$, by

(1) $$D_{\mathrm{LOS}}=\left[\begin{array}{ccc} \sin\left(\theta \right)\cos\left(\alpha \right) & -\sin\left(\theta \right)\sin\left(\alpha \right) & -\cos\left(\theta \right) \end{array}\right]\left[\begin{array}{c} D_{E}\\ D_{N}\\ D_{U} \end{array}\right]$$

The row vector is defined by the incidence angle θ and the azimuth of the satellite track α (Wright et al., 2004). It specifies the components of the vector p̂ $=\left(p_{x},p_{y},p_{z}\right)$ pointing from a point on the ground to the satellite and thus determining the proportions of eastward, northward and vertical displacement in the LOS velocity. Similarly, the LOS displacement of each point can be defined by

(2) $$D_{\mathrm{LOS}}=p_{x}D_{E}+p_{y}D_{N}+p_{z}D_{U}\;$$

Since Equations 1 and 2 contain three unknowns, at least three data sets are required to solve for the displacement vector D̂ containing the east $\left(D_{E}\right)$, north $\left(D_{N}\right)$, and vertical $\left(D_{U}\right)$ components of displacement. Since the north‐south velocities are well constrained by GNSS data, we assume that $D_{N}=GNSS_{\mathrm{north}}$ and subsequently constrain the N‐S component by a smoothed interpolated GNSS velocity field. A least squares inversion is used to solve for D̂, following the general equation

(3) $$\left[\begin{array}{c} LOS_{D}^{\mathrm{036}}\\ LOS_{D}^{138}\\ LOS_{A}^{131}\\ LOS_{A}^{058}\\ GNSS_{\mathrm{north}} \end{array}\right]=\left[\begin{array}{ccc} p_{x}^{036} & p_{y}^{036} & p_{z}^{036}\\ p_{x}^{138} & p_{y}^{138} & p_{z}^{138}\\ p_{x}^{131} & p_{y}^{131} & p_{z}^{131}\\ p_{x}^{058} & p_{y}^{058} & p_{z}^{058}\\ 1 & 0 & 0 \end{array}\right]\times \left[\begin{array}{c} D_{E}\\ D_{N}\\ D_{U} \end{array}\right]$$

The study area is covered by Sentinel‐1 ascending tracks 058 and 131 and descending tracks 036 and 138. Accordingly, the number of InSAR data sets in the matrix varies between two and four, using the maximum of available look angles for each pixel. The north component $p_{x}$ is constrained with GNSS velocities, thus $D_{N}$ is effectively removed from the system and the resulting displacement vector contains the E‐W and vertical deformation rates for each pixel. Since we work with displacement velocities, in the following we will refer to the E‐W, N‐S, and vertical components of deformation as $V_{e}$, $V_{n}$, and $V_{u}$, respectively.

## 4 Vertical Deformation Field and Relative Fault Activity

### 4.1 Regional Trends

The vertical deformation field broadly shows uplift in the northern parts of the study area, whereas the central and southern region, especially the region around the Büyük Menderes Graben, are dominated by subsidence of several mm/yr (maximum −12 mm/yr; Figure 4).

**Figure 3:**
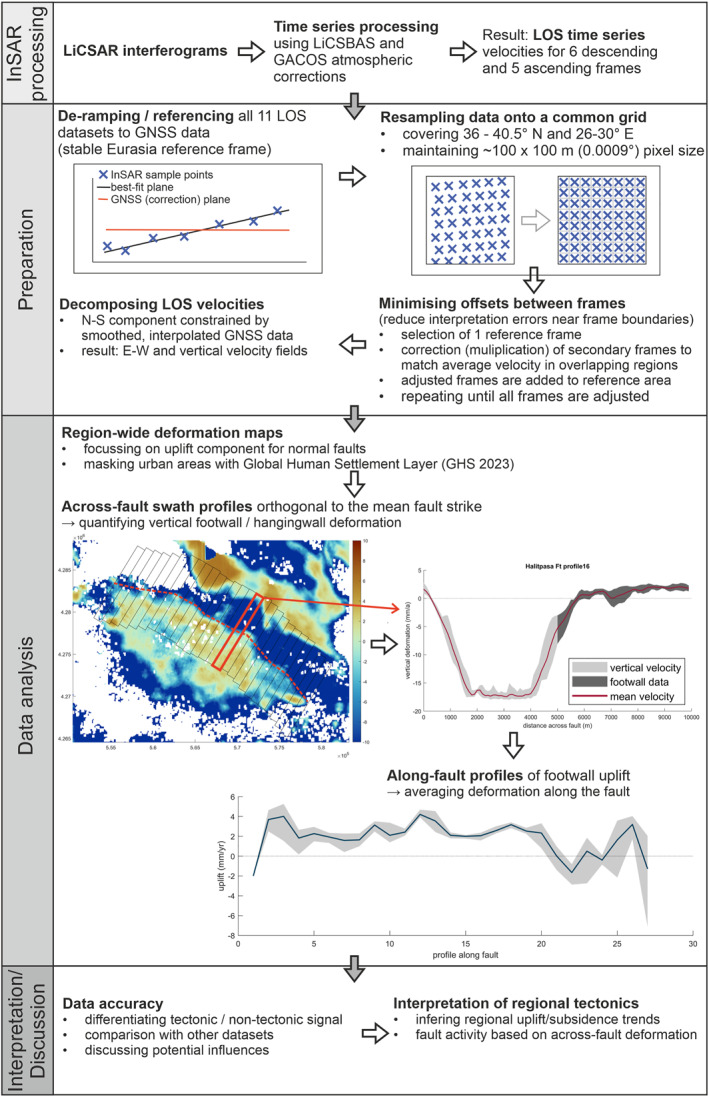
Schematic diagram of the processing, data analysis, and interpretation in this study, suitable as a generalized workflow for investigation of normal fault networks using InSAR time series.

**Figure 4:**
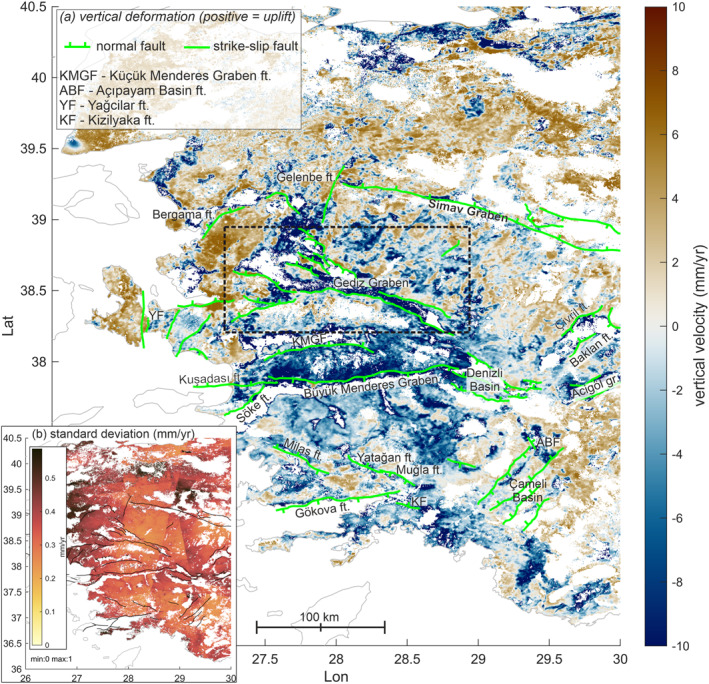
(a) Vertical velocity field and simplified fault network (green lines). (b) Standard deviation.

Several, but not all active faults show a difference in vertical deformation across mapped fault traces, with uplift in the footwall and subsidence in the hangingwall. Faults situated in subsiding regions, most notably the BMGF, show a difference in deformation expressed by slow subsidence rates in the footwall and faster subsidence rates in the hangingwall. The fastest deformation is observable at fault zones in the Gediz Graben. Other faults showing active deformation are, for example, the Pamukkale fault in the Denizli basin, the Çivril, Baklan, and Acigöl graben faults in the north‐east and the Gökova and Söke faults in the south. Uplift rates were determined for all faults that show a clear tectonic deformation signal approximately along the mapped fault traces (Table 2). These rates are not corrected for regional uplift or subsidence trends, resulting in negative footwall uplift for some faults. Faults with unclear or weak uplift signal are, for example, the Muǧla, Yataǧan, Babadaǧ, and Kuşadası faults. Faults in the İzmir region, which have a notable strike‐slip component, mostly show very little vertical deformation (with the exception of the Yaǧcilar ft.) similar to faults in the Çameli basin. We also investigated several faults with uncertain Holocene activity, the Gelenbe fault north of the Gediz Graben and the Kızılyaka fault east of the Gulf of Gökova. The Gelenbe fault, which potentially ruptured in a series of moderate earthquakes in 2020 (see Section 2.3), shows an unclear morphological trace and no detectable surface deformation, which could be attributed to its strike‐slip kinematics. The Kızılyaka fault is only featured in the NOAFaults catalog (Ganas et al., 2013) and features a small topographic escarpment, but also shows no surface deformation. In contrast, the Küçük Menderes Graben features notable uplift along the northern margin, where no active fault is known to date, as well as a weak signal at the southern margin, which likely hosts the Küçük Menderes Graben Fault, though its recent activity is not clear (Seyitoğlu & Işık, 2009). Several faults which are clearly active, such as the BMGF, show slow active surface deformation. Note that uplift rates can be influenced by regional uplift or subsidence and thus are not directly convertible to fault throw/slip rates.

**Table 2:** Footwall Uplift Rates of Faults With Clear Tectonic Uplift Signal, Determined From Across‐Fault Swath Profiles

Fault	Mean (mm/yr)	Max. (mm/yr)	Comments
Gediz Graben Region:			
Menemen ft.	5.93	6.58	
Akhisar ft.	2.10	5.20	
Akselendi ft.	3.73	5.35	
Gölmarmara ft.	−2.99	2.72	Deformation only on eastern segment
Ozanca ft.	2.87	6.01	2017 $M_{W}$ 5.1 source
Halitpaşa ft.	1.82	4.21	
Manisa ft.	3.28	6.30	
Kemerdamlari ft.	3.43	6.38	
Killik ft.	1.83	6.24	
GGBF	1.84	6.31	
KMGF	−2.67	1.92	
Denizli Basin:			
Pamukkale ft.	0.34	2.68	
Kaleköy ft.	−0.33	1.92	Name after (Koçyiğit, 2005)
Honaz ft.	0.97	2.39	
Aşaǧidaǧdere ft.	0.16	1.48	
Eastern Region:			
Çivril ft.	2.29	4.01	
Baklan ft.	1.65	3.60	
Maymundaǧ ft.	−2.15	1.57	
Acigöl ft.	2.66	4.35	Probably 2019 $M_{W}$5.9 source
Acıpayam Basin ft.	2.13	3.99	2019 $M_{W}$5.8 source (Yang et al., 2020)
Southern Region:			
Söke ft.	1.19	3.82	
Milas ft.	1.38	3.44	Clear deformation only on western segment
Yataǧan ft.	0.39	3.28	
Gökova ft.	1.54	4.60	

*Note.* Maximum is the fastest uplift rate of all profiles, mean is the average of maximum velocities from all profiles. See Figures 5 and 10 for fault locations. Uplift rates reflect relative fault activity but may be affected by regional uplift or subsidence (causing negative values) and cannot be easily converted into fault throw rates.

### 4.2 Characteristics of Active Fault Zones

#### 4.2.1 Gediz Graben

The dominant tectonic structure of the Gediz (Alaşehir) Graben is the detachment fault at the southern side, which has been active since Miocene, exhuming the Menderes Massif. It forms a low‐angle detachment dipping ∼ 15–30° to the north and is believed to be inactive, while the active high‐angle Gediz Graben Bounding Fault (GGBF) formed in its hangingwall (Bozkurt & Sözbilir, 2004; Çiftçi & Bozkurt, 2009; Gessner et al., 2001; Purvis & Robertson, 2004; Seyi̇toğlu et al., 2002). According to Buscher et al. (2013), the transition from faulting at the low‐angle detachment to the high‐angle fault zone occurred between the late Pliocene and early Quaternary. The GGBF consists of three segments, the eastern Alaşehir segment, the central Salihli segment and the western Turgutlu/Armutlu segment. Naming and mapped fault traces, especially for the western segment and adjacent faults, vary in literature. Kent et al. (2016) used cross‐sections interpreted from published seismic and outcrop maps and the relationship between throw and relief to determine the long‐term slip rates along the GGBF. Throw rates (vertical part of slip rates), are in the range of 0.4–1.3 mm/yr (Kent et al., 2016), accelerating to up to 2.0 mm/yr at about 0.6–1 Ma (Kent et al., 2017). The northern side of the graben hosts the antithetic Killik and Kemerdamları Faults. In the west, the graben splits up into several sub‐basins hosting multiple active faults.

**Figure 5:**
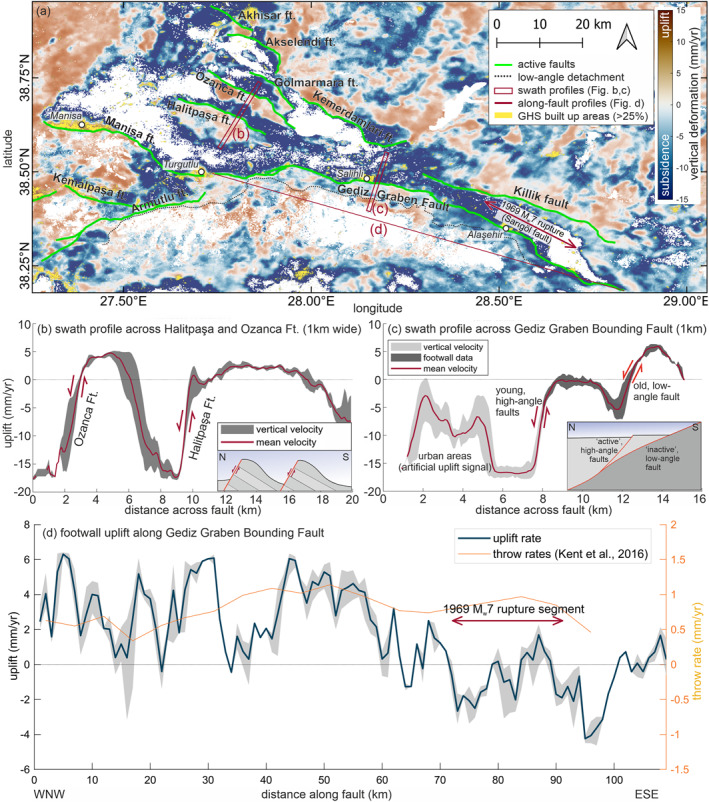
Vertical deformation in the Gediz Graben. (a) Vertical deformation field and mapped faults (simplified). Red boxes outline swath profiles (b and c), red line indicates the along fault profile (d). Yellow areas show built‐up areas with more than 25% land coverage based on the Global Human Settlement Layer (GHS, Pesaresi and Politis (2023)). Urban areas show a clear correlation with false uplift signals. (b) Swath profile across the Halitpaşa and Ozanca Ft., showing clear uplift; inset depicts cartoon of block tilt. (c) Swath profile across the Gediz Graben Bounding Fault (GGBF), showing deformation at both the old, low angle, and the young, high angle fault splays. (d) Along‐strike profile of the GGBF footwall, from 109 across‐fault swath profiles. Note the contrast in uplift rates between the 1969 rupture segment and the central/western part of the fault. Throw rates from cross‐section analysis (Kent et al., 2016) vary between ∼ 0.3 and 1.1 mm/yr, with the fastest rates in the center of the fault.

Figures S5–S7 in Supporting Information S1 show detailed analyses of deformation along other faults in the Gediz Graben from across‐fault swath profiles. The Ozanca, Akselendi, Kemerdamları (S5), Gölmarmara (S6), and Halitpaşa Faults (S7) all exhibit a consistent deformation signal across most or the entire length of their respective mapped fault traces. Deformation is quantified using 1 km wide swath profiles across the fault traces, perpendicular to the mean fault strike (see Figures 3 and 5c). For robust quantification of the maximum uplift, we first take the average of all values along the profile (red line in Figure 5), then we determine the maximum of this within the footwall (dark gray) of the profile. The maximum value (gray shaded) of the red curve and the spread of values at this point are used to create along‐fault uplift profiles (Figure 5d). Assuming that the slip distribution of the observed tectonic deformation is comparable to long‐term fault slip, a triangular, or elliptical slip distribution would be expected, with the maximum slip in the fault center, decreasing toward the tips (Cowie & Roberts, 2001; Manzocchi et al., 2006; Roberts, 2007; Schlagenhauf et al., 2008).

#### 4.2.2 Çivril and Acıgöl Grabens

**Figure 6:**
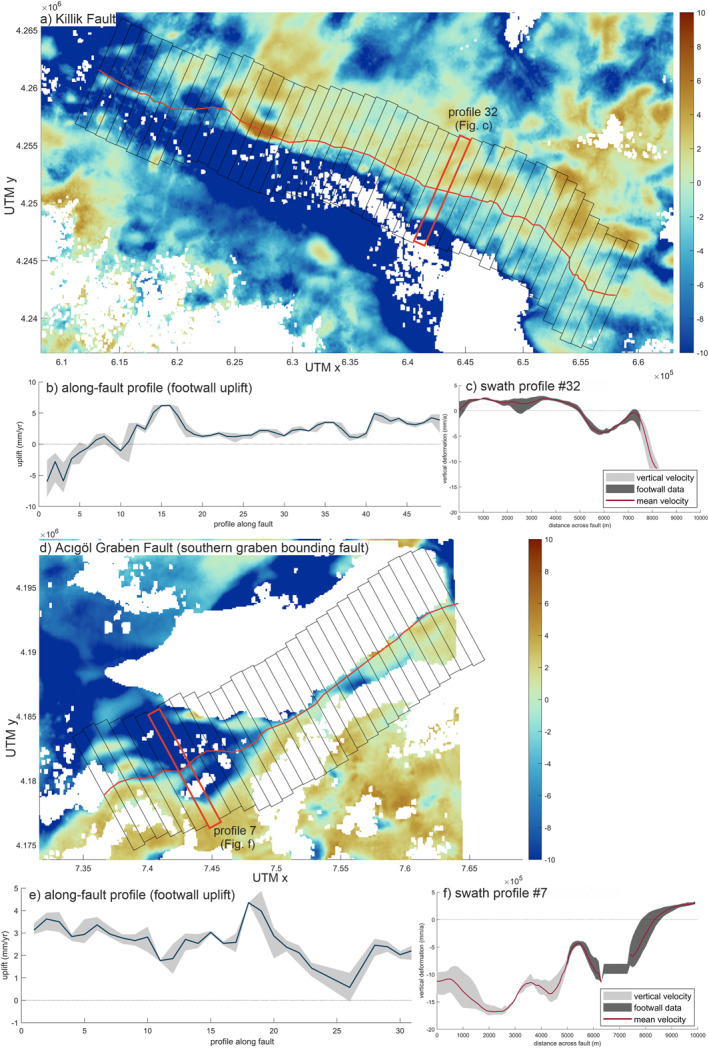
Detailed study of active deformation at the (a) Killik Fault (Northern Gediz Graben boundary) and the (d) Acıgöl Fault (Southern Acıgöl Graben boundary) from averaged footwall uplift along the fault trace (b and e) based on across‐fault swath profiles (c and f) of the vertical deformation.

The Çivril and Acıgöl Grabens are both bounded by active normal faults on either sides, all of them showing a clear deformation signal along the entire length of the mapped fault trace (Figure 6; Figures S7 and S8 in Supporting Information S1), namely the Çivril Ft., Baklan Ft., Acıgöl Ft., and Maymundağ Ft. The western part of the Maymundağ Ft. is undergoing subsidence in its footwall though this area has been affected by the 2019 earthquake in the Acıgöl Graben. The antithetic Acıgöl Fault, likely the source of the 2019 earthquake, exhibits a secondary deformation front in the western segment, where the main fault (and mountain front) bends toward south. This is also visible in the topography as a small escarpment.

Most other structures in the WAEP show less consistent deformation signals along the mapped fault traces.

## 5 Interpretation and Discussion

### 5.1 Vertical Deformation

#### 5.1.1 Accuracy of Velocity

The propagated standard deviation of our inverted vertical velocities (Figure 4b) is in the range of 0–1 mm/yr, though the real deviation could be larger. We compare our vertical velocity rates with those determined by other studies. Poyraz et al. (2019) and Poyraz and Hastaoğlu (2020) determined deformation rates in the eastern Gediz Graben from GNSS and persistent scatterer InSAR (PSInSAR). Vertical deformation from multiple sensors is also available for several coastal locations (Erkoç et al., 2022); however, comparison with these data is less accurate owing to decorrelation in the coastal areas (see Supplement 2). Briole et al. (2021) provide vertical GNSS velocities for stations in Greece and Western Türkiye.

**Figure 7:**
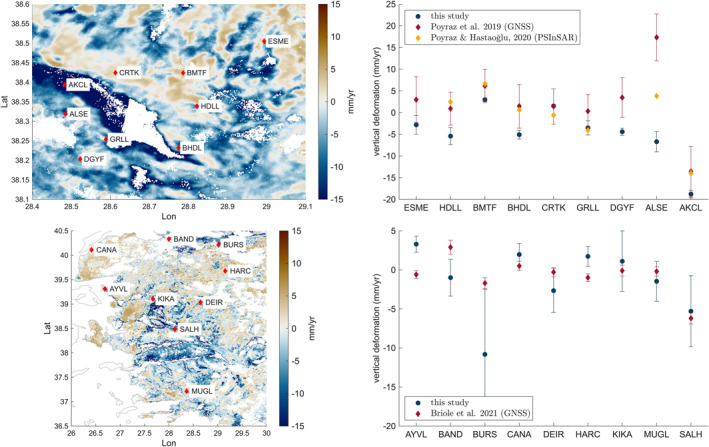
Top: Comparison of vertical deformation rates from InSAR (this study) to GNSS (Poyraz et al., 2019) and persistent scatterer InSAR (Poyraz & Hastaoğlu, 2020) for nine locations in the eastern Gediz Graben. Bottom: Comparison of vertical InSAR deformation rates to GNSS data by Briole et al. (2021). 1σ error bars.

For comparison with other studies, we average InSAR velocities in a square of ∼1×1 km for the local studies (Poyraz & Hastaoğlu, 2020; Poyraz et al., 2019) and 2×2 km for the region‐wide data (Briole et al., 2021) around the sites of the other studies. As before, we masked the InSAR velocity field with built‐up areas with more than 5% of surfaces covered by buildings (GHS 2023), effectively removing urban areas. We excluded stations with less than 30% of the square area covered by InSAR data, thus excluding most stations located in cities. Error bars (Figure 7 and supplement 4) display the standard deviation of the averaged velocities within the defined areas.

InSAR velocities are generally lower (more negative) than the GNSS and PSInSAR velocities determined by Poyraz et al. (2019) and Poyraz and Hastaoğlu (2020) at most locations (Figure 7). If GNSS/PSInSAR velocities are more accurate than InSAR, and the observed differences are representative for the entire study region, uplift rates could be systematically underestimated in our study by several mm/yr. For the broad deformation field, this would make a minor difference, however slip rates at individual faults would be faster than the rates we observe. Comparison with GNSS velocities by Briole et al. (2021) shows notable discrepancies at few locations with no generalized trend observable. The direct comparison however is complicated because InSAR velocities represent averaged deformation rates for a larger area, and even small errors on station coordinates or differences in observation intervals lead to unavoidable discrepancies between both data sets. The comparison with PSInSAR deformation rates is probably more reliable than comparison with GNSS data, considering the large uncertainties in vertical GNSS deformation rates (most GNSS studies do not publish vertical deformation rates at all). Nevertheless, deformation rates should be interpreted in a broader regional context, considering possible local uncertainties, for example, by deducing fault‐specific deformation from observations along the entire length of the fault.

Short term deformation rates (GNSS, InSAR, PSInSAR) are ∼ 5 times faster than long‐term rates from cross‐section profiles or river profile analysis (Kent et al., 2017, 2020) and ^36^Cl‐dating (Mozafari et al., 2022), depending on the conversion from uplift to throw/slip rates. Long‐term slip rates average deformation over multiple seismic cycles, whereas InSAR velocities represent short‐term movements within the interseismic period. Accordingly, uplift rates and respective throw rates of faults from InSAR are not necessarily representative of the long‐term throw rates and should not be used for modeling interseismic deformation or estimating earthquake recurrence times.

#### 5.1.2 Tectonic Versus Non‐Tectonic Signals

**Figure 8:**
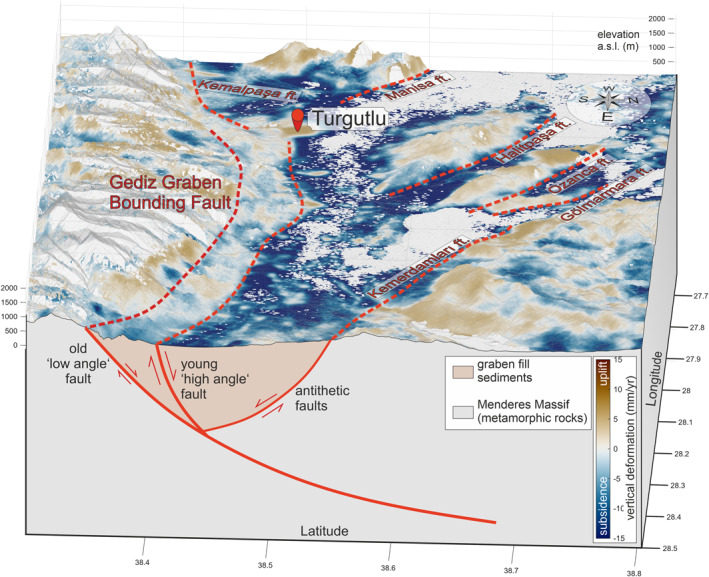
View of the central Gediz Graben toward West with vertical deformation rates draped over the ALOS‐2 DEM (2x vertically exaggerated). The young, “high‐angle” Gediz Graben Bounding Fault is assumed to root into the older, “low angle” detachment fault at depth. Both strands show a notable difference of vertical deformation across the mapped fault trace. Deformation across the young graben bounding fault appears significantly faster along most of the fault.

On a large scale (Figure 4 and LOS‐velocity maps in Supplement 1), major morphological features are visible in the velocity field, suggesting a relationship between topography and deformation rates. This is mostly due to the rapid subsidence observed in the basins. On direct comparison of the topography, lithology, vertical deformation signal, and location of active fault zones, this correlation is not consistent. In the Gediz Graben (Figure 8), a clear contrast in uplift/subsidence rates can be observed along most of the active graben bounding fault. The fault zone spatially correlates with the topographic margin of the basin, though the lower slopes of the mountain front are relatively shallow. While the hangingwall subsidence is mostly due to anthropogenic effects, the footwall is uplifting along most of the fault. Similarly, the topographic contrast between footwall and hangingwall at many of the fast‐deforming faults, for example, the Halitpaşa Fault in the Gediz Graben, is relatively small, thus the clear deformation signal along the fault scarp is less likely to be due to topographic or atmospheric effects.

Accordingly, where the uplift signal correlates spatially with fault traces, the observed deformation appears to be predominantly due to tectonic processes. Nevertheless, some topographic features, such as the western boundary of the Manisa Basin, are not controlled by active faults but still exhibit a noticeable deformation signal, thus topographic effects need to be considered in interpretations. Similarly, the Yaǧcılar strike‐slip fault shows a surprisingly fast deformation signal which cannot be attributed to tectonic movements. Our data generally agree with the non‐tectonic deformation signals found by other studies. However, as these almost exclusively focus on the subsidence in grabens, we are unable to confirm deformation rates due to large uncertainties and decorrelation of the InSAR velocities in the grabens.

**Figure 9:**
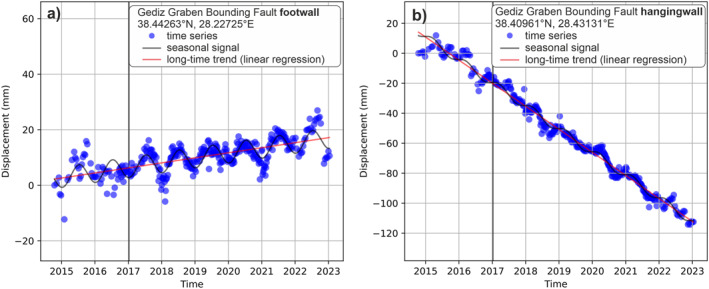
Example of footwall (a) and hangingwall (b) time series at the Gediz Graben Bounding Fault (descending frame 138D_05142). Both footwall and hangingwall deformation show a seasonal signal, which is exceeded by the long term uplift or subsidence trend.

We find a seasonal signal in almost all locations, including the footwall of active faults. However, the long‐term (8–9 year) deformation exceeds the seasonal variations. Figure 9 shows typical time series from the footwall (a) and hangingwall (b) of the GGBF. Despite the seasonal variations, the long term trends show clear subsidence in the hangingwall and uplift in the footwall of the fault.

#### 5.1.3 Strain Distribution in the Gediz Graben

We picked the Gediz (Alaşehir) Graben for a detailed study of local/regional strain distribution due to the abundance of well‐mapped, active faults, its structural complexity and available data and literature. Detailed analyses reveal insights into several commonly misinterpreted characteristics of regional faulting; First, vertical deformation is not focused on the southern side of the graben, which hosts the main graben bounding fault. While all faults in the graben show vertical deformation, the antithetic faults in the north (Kemerdamları and Killik Ft.) in some parts appear to be moving faster than the GGBF. In the graben center, the Halitpaşa and Ozanca faults are uplifting and tilting smaller blocks at ∼ 3–5 mm/yr (Figure 5b). Second, the southern graben margin shows two deformation fronts (Figures 5c and 8). These correlate with the locations of the active GGBF in the north and the low‐angle detachment fault in the south, suggesting that the Gediz detachment, contrary to established opinions (Buscher et al., 2013; Gessner et al., 2001; Seyitoğlu et al., 2002), might still be active. However the contrast in vertical deformation along the southern, “low angle” fault is less pronounced. Additionally, effects of topography, vegetation, and land use need to be considered as the fault is located further from the active basin margin and displaces the basin fill deposits in the hangingwall against the metamorphic rocks of the Menderes Massif. The amount of uplift expected on a shallow (15‐30°) dipping normal fault, even under relatively fast deformation rates is very small. Further studies incorporating N‐S displacement, for example, using precise GNSS measurements would be needed to verify this process.

Third, deformation at the GGBF (Figure 5d) is faster on the western and central segments with up to ∼ 6 mm/yr uplift, but comparably slow on the eastern (Alaşehir) segment, which ruptured in a $M_{W}$ 6.9 earthquake in 1969 (Arpat & Bıngöl, 1969; Eyidoğan & Jackson, 1985). The Manisa Ft. shows a fast uplift of ∼ 5–6 mm/yr along most of its length, exceeding long‐term slip rates of <0.3 mm/yr based on ^36^Cl‐dating (Mozafari et al., 2022). The eastern part of the mapped fault, connecting to the GGBF, shows no active deformation.

To conclude, we find that strain in the Gediz Graben is distributed across multiple active faults, which is also supported by the distributed seismicity across the graben. While the GGBF is the structurally and morphologically most prominent fault zone, antithetic faults at the northern graben margin and secondary faults in the graben center likely accommodate a notable portion of the regional extension. The old detachment fault might still accommodate some deformation though this would need further studies to verify and probably makes up only a small fraction of the regional deformation. It is likely that deformation in other grabens and across the region is distributed similarly.

#### 5.1.4 Vertical Deformation Rates

**Figure 10:**
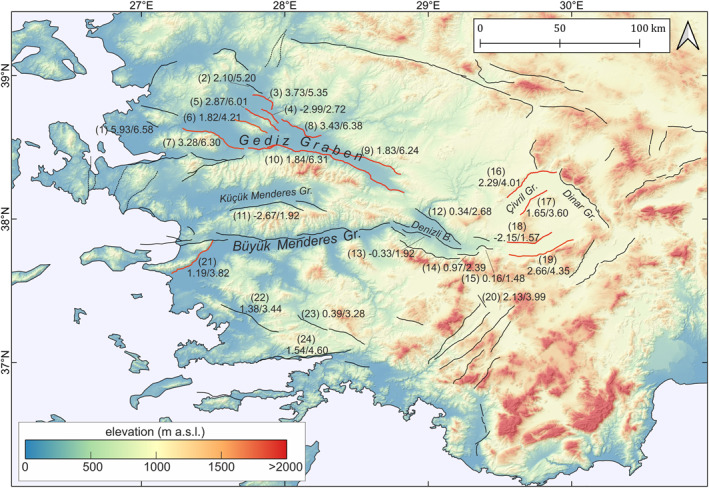
Overview of footwall uplift rates of faults throughout the Western Anatolian Extensional Province (mean uplift/maximum uplift (mm/yr)). Uplift rates were only determined for faults with a clear uplift contrast between footwall and hangingwall; faults highlighted in red show a very consistent deformation signal along most of their mapped fault traces (also see Figure 6; Figures S5–S8 in Supporting Information S1). Note that given uplift rates may be influenced by regional uplift/subsidence trends. Dashed lines show strike‐slip faults. (1) Menemen ft., (2) Akhisar ft., (3) Akselendi ft., (4) Gölmarmara ft., (5) Ozanca ft., (6) Halitpaşa ft., (7) Manisa ft., (8) Kemerdamları ft., (9) Killik ft., (10) Gediz Graben Bounding Fault, (11) Küçük Menderes Graben Fault (12) Pamukkale ft., (13) Kaleköy ft., (14) Honaz ft., (15) Aşaǧidaǧdere ft., (16) Çivril ft., (17) Baklan ft., (18) Maymundaǧ ft., (19) Acıgöl ft., (20) Acıpayam Basin ft., (21) Söke ft., (22) Milas ft., (23) Yataǧan ft., (24) Gökova ft.

Footwall uplift along active faults in the study region (Figures 5 and 10, Table 2) varies significantly. Most of the known active faults, especially faults in the Gediz Graben, but also the Çivril, Baklan, and Acıgöl faults in the north‐eastern region, and some faults in the southern part of the WAEP show a clear tectonic deformation signal along the mapped fault traces. However, few active faults, such as the Büyük Menderes Graben Fault, the Simav Graben fault, or the Muǧla fault, show little to no detectable deformation signal. Fault slip rates vary over a range of timescales, influenced by fault linkage, interaction with other faults, and earthquake clustering (Cowie & Roberts, 2001; Friedrich et al., 2003; Mildon et al., 2022). Therefore we suggest that these faults with no detectable deformation signal should not be considered inactive on the basis of our study, and instead we hypothesize that these faults could be undergoing a period of lower deformation/slip rate, or other factors contributing to the InSAR signal could disturb the observations.

Detailed investigation of active faulting in the Gediz region (Figure 5) further highlights the following aspects relevant to the regional seismic hazard: (a) The antithetic faults (Killik and Kemerdamları faults), commonly considered less important, show faster deformation over the studied time period than the main GGBF and therefore should be considered as equal seismic hazard potential. (b) The old, low‐angle detachment fault, contrary to established belief, appears to be active as there is a change in uplift rate coincident with the fault trace. (c) The GGBF has spatial correlation between current deformation rate and the 1969 $M_{W}$ 6.9 earthquake on the Alaşehir segment, which is not observable in long‐term throw rates of the fault segments (Kent et al., 2017). We interpret that the reduced deformation rate at the ruptured segment relates to stress released due to the earthquake. If correct, this would imply that the other segments are still stressed, and potentially capable of producing a damaging earthquake of similar magnitude.

### 5.2 Tectonic Implications

#### 5.2.1 Regional Uplift and Subsidence Patterns

Figure 4 shows regional uplift in the northern and eastern parts of the study area, as well as localized areas of uplift along the Aegean coast. The central part of the region, hosting the Menderes Massif and the most prominent graben systems, is undergoing rapid subsidence in the range of up to −12 mm/yr. This is mostly consistent with vertical deformation rates derived by Weiss et al. (2020) though they find less rapid extension across the Menderes Massif region. Even considering a possible tendency of our data toward subsidence, as observed in comparison with data by Poyraz et al. (2019) and Weiss et al. (2020), subsidence persists across this region. The fastest subsidence rates outside the grabens are observed in the Aydın range, north of the Büyük Menderes Graben, and independent of topography or anthropogenic impacts. Large‐scale uplift models by McNab et al. (2018) show indicators of uplift across entire Türkiye since Miocene, with recent uplift rates of 0–0.2 mm/yr for westernmost Türkiye.

In a geodynamic context, the observed subsidence can be partly explained with the rapid extension across these graben systems and related thinning of the crust. When combining long‐term regional uplift with rapid N‐S extension driving the opening of graben structures, erosion, and incision, this supports the build‐up of the regional relief, with elevation differences of over 2,000 m between the grabens and the peaks in the Bozdağ mountain range.

Nevertheless, direct comparison with uplift rates by McNab et al. (2018) is not possible due to the vastly different time frames, scale, and spatial resolution of both studies.

#### 5.2.2 Fault Deformation Rates

Figure 10 shows the footwall uplift rates measured across the WAEP. Uplift rates are only given for faults with clearly identifiable contrast between hangingwall and footwall deformation rates. As discussed above, observed uplift rates may be biased by other influences. To control topographic bias, faults are plotted on digital elevation data. It is not possible to derive accurate fault‐slip rates from the measured uplift rates, since the hangingwall subsidence cannot be quantified.

The WAEP is actively extending in N‐S direction with about 20 mm/yr (Aktug et al., 2009). When using the observed uplift rates, and a notable contrast in uplift rates between the footwall and hangingwall of a fault, as a proxy for fault activity, the results clearly indicate that deformation is accommodated across a large number of faults of varying size.

#### 5.2.3 Block Versus Continuum Deformation

Early GPS studies (McClusky et al., 2000; Nyst & Thatcher, 2004; Reilinger et al., 2006) have described the deformation of Anatolia and its surroundings using block models. These were characterized by relatively large blocks, with most block boundaries along actual plate or microplate boundaries, such as the NAF or the Hellenic Arc. In Reilinger et al.’s (2006) block model, the WAEP is divided by two block boundaries, one approximately along the Denizli basin and Gediz Graben system, and the second roughly following the Fethiye‐Burdur‐Trend. Nyst and Thatcher (2004) created a block model with similar block boundaries, though here the main block boundary dividing the northern from the southern part of the WAEP is modeled along the Denizli Basin and Büyük Menderes Graben and continuing north‐westward through the İzmir region, approximately orthogonal to the known mapped faults. Özdemir and Karslıoğlu (2019) were able to approximately reproduce block models by these earlier studies, though again with varying block boundaries and varying numbers of blocks. The idea of block boundaries segmenting western Anatolia has been re‐iterated by İnan et al. (2012), claiming that the absence of earthquake precursory anomalies could be explained by block boundaries acting as strain barriers, though this remains speculative. More recently, Ergintav et al. (2023) made a simple block model, finding relatively little strain within the blocks and large strain along major fault zones, though this is predominantly observed in eastern Anatolia, where the EAFZ and NAFZ are dominating the strain field. In their block model, the majority of the WAEP is placed within a single block bounded by the Simav‐Sultandaǧi fault system in the north, and the Milas fault, extended to the east and west, in the south.

Contrary to other early GPS studies, Aktug et al. (2009) found that strain rates in the interiors of proposed blocks in Western Anatolia are too high to justify a block‐like behavior. Based on multiple detailed block models they conclude that block models can only explain the observed deformation when blocks are made so small that it essentially becomes a continuum. Results from our InSAR time series support this concept, indicating that deformation is distributed across a large number of active faults of varying size and slip rates (Figure 10). Regions without mapped active faults are found only in few places, such as between the Gediz Graben fault system and the Simav Graben.

The number of blocks and the location of proposed block boundaries varies between each published model and block boundaries oppose knowledge of existing fault zones. Depending on the model, major active graben systems might be placed in the interior of blocks (Reilinger et al., 2006), despite multiple large earthquakes proving rapid long‐term deformation. We conclude that continuum deformation is more accurate to describe the tectonics of the WAEP, whereas block‐like behavior is likely restricted to larger tectonic units. Detailed block models might identify zones of elevated strain rates and help describing the deformation field, while large scale block models provide a plausible description of relative plate and microplate motions.

### 5.3 Quantifying Deformation in Extensional Regimes

While a large quantity of studies focuses on strike‐slip fault zones, such as the North Anatolian Fault (Cakir et al., 2005; Hussain et al., 2018; Walters et al., 2011), InSAR time series is not routinely applied to extensional regimes. Here we discuss practical approaches, as well as capabilities and limitations to quantify deformation in actively extending regions with Sentinel‐1 InSAR time series.

Horst‐graben systems, and their typical morphological expression of alternating flat, sediment‐filled basins and uplifted mountain chains, are a typical structure of actively deforming extensional regimes. Accordingly, the associated problems caused by topographic effects and differential land use are not unique to this study location. We find that quantifying the footwall uplift can be used as a proxy for fault activity. The use of systematic across‐fault swath profiles reduces interpretation bias and the impact of outliers. Urban areas can cause strong false deformation signals but can be easily identified and masked using world‐wide available data sets, such as the Global Human Settlement Layer (here we use the built‐up surfaces layer (Pesaresi & Politis, 2023)). For fault‐specific analyses, earthquake catalogs should be consulted to identify earthquakes within the observation period, to distinguish interseismic and coseismic deformation. All results should be validated with field observations, digital elevation data, or other satellite imagery.

Despite this approach aiming to eliminate uncertainties and relying on the most useful parts of the deformation signal, several limitations must be considered for interpretation of tectonic processes. First, this approach cannot be used to derive fault‐slip rates, due to the unknown subsidence component. The hangingwall subsidence is probably greater than the footwall uplift, so most of the deformation signal is lost when only looking at the footwall uplift. Second, the accommodation of regional extensional strain cannot be quantified, though this would be a valuable metric to assess the tectonic processes. For none east‐west orientated fault networks the E‐W component could be used to quantify this. Third, some faults, such as the Büyük Menderes Graben fault, show a notable difference between hangingwall and footwall deformation, though even the footwall deformation is negative (subsiding). When looking only at the footwall vertical deformation, this might result in underestimates of the fault activity. Finally, vegetation, different land use, and topographic/atmospheric biases are dominant in the hangingwall, but also influence the footwall deformation signal of faults. While signals induced by built‐up areas can be easily removed by masking the affected areas with available data sets, as described above, biases due to vegetation and topography can only be roughly estimated using other available data. Advanced methods to distinguish tectonic and non‐tectonic influences are needed here to allow more robust quantification of fault deformation.

## 6 Conclusions

The overarching objective of this study is to investigate the deformation field of the WAEP with respect to active normal faults. We demonstrate that vertical deformation can be used to estimate the relative activity of faults/branches and present a workflow suitable to derive meaningful results in normal‐faulting dominated regions.

Regional fault activity varies both spatially and temporally. We observe surface deformation at the majority of faults in the WAEP, with apparent footwall uplift up to ∼ 5 mm/yr. Regional strain is not localized on the major graben‐bounding fault zones. Instead, deformation is distributed across a large number of faults with varying size and morphological expression, including antithetic and secondary faults within the grabens and possibly faults that were previously not inferred to be active, such as the low‐angle normal faults along the Gediz Graben. Finally, we show a possible correlation between active deformation at the Gediz Graben boundary faults and the 1969 rupture with potential implications for seismic hazard of the other segments. For future studies using InSAR time series in extensional settings, improved techniques to separate the topographic influence on the time series signal from actual tectonic deformation will be required. This could significantly enhance the robustness of interpreted activity and deformation rates.

## Supporting information

Supporting Information S1

Supporting Information S2

## Data Availability

All used interferograms are available via the COMET LiCS portal at https://comet.nerc.ac.uk/comet‐lics‐portal/ (Lazecký et al., 2020) and were processed with the open‐source software LiCSBAS, available via https://github.com/yumorishita/LiCSBAS (Morishita et al., 2020). The vertical velocity field is attached in Supporting Information S2.
