# Supplementary material for: Active Deformation Across the Western Anatolian Extensional Province (Türkiye) From Sentinel‐1 InSAR
Source: Tectonics. 2024 Nov 24;43(11):e2023TC008086. doi: 10.1029/2023TC008086 (PMC11586515; doi:10.1029/2023TC008086)
Supplement: Supplementary file 1 — Supporting Information S1 [file TECT-43-0-s002.pdf]

Supporting Information for "Active Deformation Across the Western Anatolian Extensional Province (Türkiye) from Sentinel-1 InSAR"

Manuel Diercks[1], Ekbal Hussain[2], Zoë K. Mildon[1], Sarah J. Boulton[1], Milan Lazecký[3]

[1]School of Geography, Earth and Environmental Sciences, University of Plymouth, Plymouth, PL4 8AA, United Kingdom

[2]British Geological Survey, Natural Environment Research Council, Environmental Science Centre, Keyworth, Nottingham, NG12 5GG, United Kingdom

[3]COMET, School of Earth and Environment, University of Leeds, Leeds LS2 9JT, UK

Contents of this file

1. Figure S1
2. Figure S2
3. Figure S3
4. Figure S4
5. Figure S5
6. Figure S6
7. Figure S7

8. Figure S8

9. Figure S9

10. Data Set S10

Introduction In S1 and S2 we present additional figures supporting the manuscript. S1 shows the line-of-sight (LOS) velocity fields for ascending and descending geometries. S2 shows a comparison of vertical velocities at several coastal locations with data from Erkoç et al. (2022).

S3 shows the differences between InSAR velocities referenced to GNSS data from Özdemir & Karslıoğlu (2019), Nocquet et al. (2012), and England et al. (2016), and velocities referenced to newly released GNSS data by Kurt et al. (2023), aiming to determine whether use of newly released data affects the results and interpretations. Average differences are 0.0262 mm/yr for ascending and 0.1090 mm/yr for descending geometry, respectively, with largest differences in the northernmost and southernmost parts of the area and close to zero differences in the central graben region. Interpretations are thus not significantly altered using different GNSS velocities. Notable differences persist in areas of overlapping frames, which can be reduced by later adjustment steps.

S4 shows examples of interferograms covering earthquakes larger than M_W 5.0 with no observable surface deformation.

S5 to S9 show detailed analyses of active faults in the WAEP which exhibit consistent deformation along most of their mapped fault length. Swath profiles of 1 km width orthogonal to the faults' strike are generated to quantify footwall uplift and determine the difference between footwall and hangingwall deformation. Along-fault profiles are then

calculated from averaged footwall uplift of individual swath profiles.

S10 is a zip-folder containing figures which provide additional information on the InSAR processing. For each frame we provide the small baseline network, mask, masked filtered velocity (mm/yr), coherence and standard deviation of the velocity (mm/yr). Additionally, we provide the vertical velocity field for the study area as a geotiff for use in future studies.

References

England, P., Houseman, G., & Nocquet, J. M. (2016). Constraints from GPS measurements on the dynamics of deformation in Anatolia and the Aegean. *Journal of Geophysical Research: Solid Earth*, 121(12), 8888-8916.

Erkoç, Muharrem Hilmi, Uğur Doğan, Hasan Yıldız, and Erdinç Sezen. (2022, October). "Estimation of Vertical Land Motion along the South and West Coast of Turkey from Multi-Sensor Observations." *Advances in Space Research*, 70(7), 1761-1772. ISSN: 0273-1177. DOI: 10.1016/j.asr.2022.06.022.

Kurt, A. I., Ozbakir, A. D., Cingöz, A., Ergintav, S., Doğan, U., & Özarpaci, S. (2023, January). Contemporary Velocity Field for Turkey Inferred from Combination of a Dense Network of Long Term GNSS Observations. *Turkish Journal of Earth Sciences*, 32(3), 275–293. https://doi.org/10.55730/1300-0985.1844

Nocquet, J. M. (2012). Present-day kinematics of the Mediterranean: A comprehensive overview of GPS results. *Tectonophysics*, 579, 220-242.

Özdemir, S., & Karslıoğlu, M. O. (2019). Soft clustering of GPS velocities from a homogeneous permanent network in Turkey. *Journal of Geodesy*, 93(8), 1171-1195.

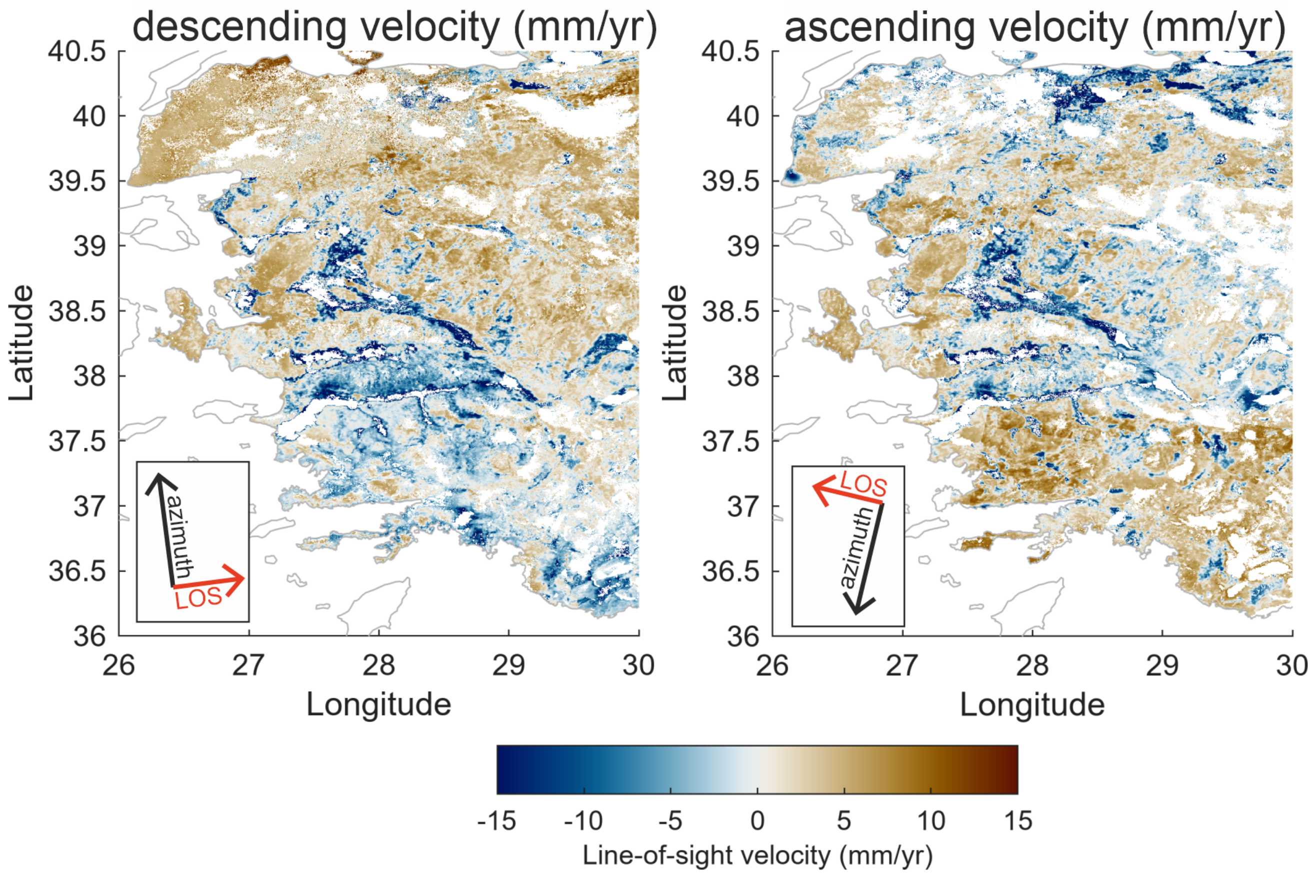

Figure S1. LOS velocity maps for ascending (a) and descending (b) tracks. Velocities were referenced to a stable Eurasia GNSS reference frame and adjusted to reduce steps between frames (see main text).

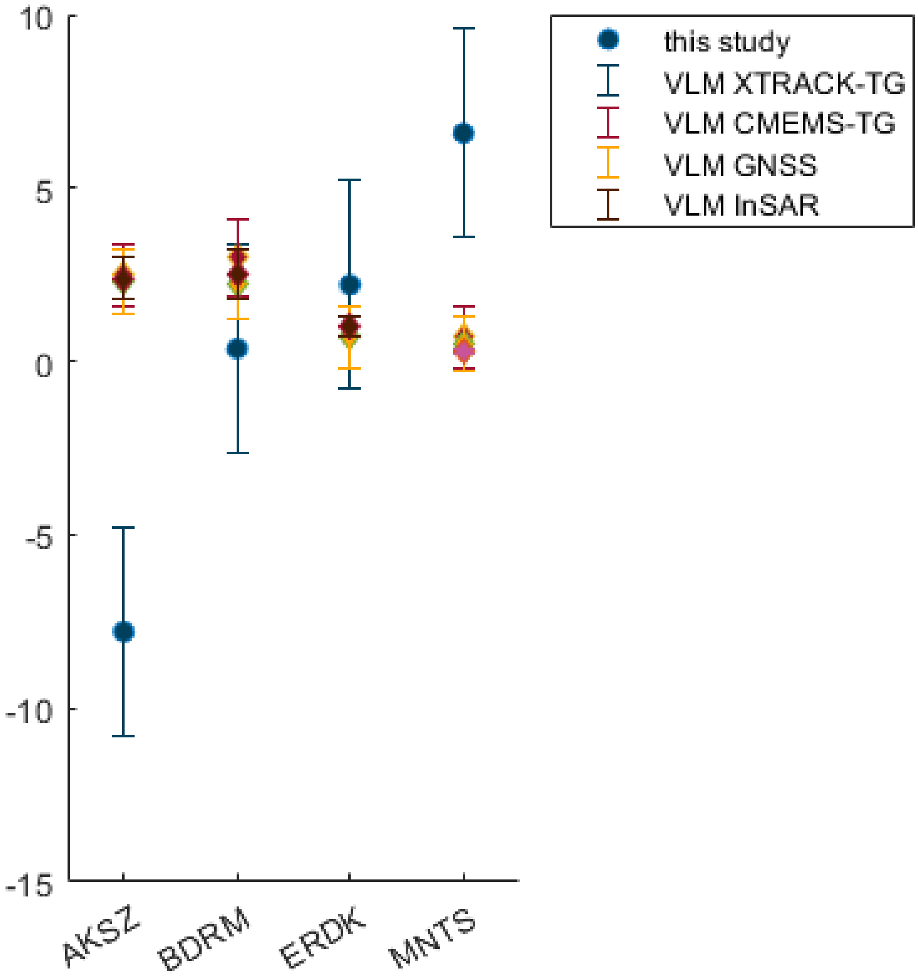

Figure S2. Comparison of vertical deformation from InSAR with multi-sensor vertical land motion from Erkoç et al. (2022). Decorrelation and noise near the coastal locations have a notable impact on the velocity field, hence the comparison shows major deviations in two of the four locations. No general trend of deviations is observable.

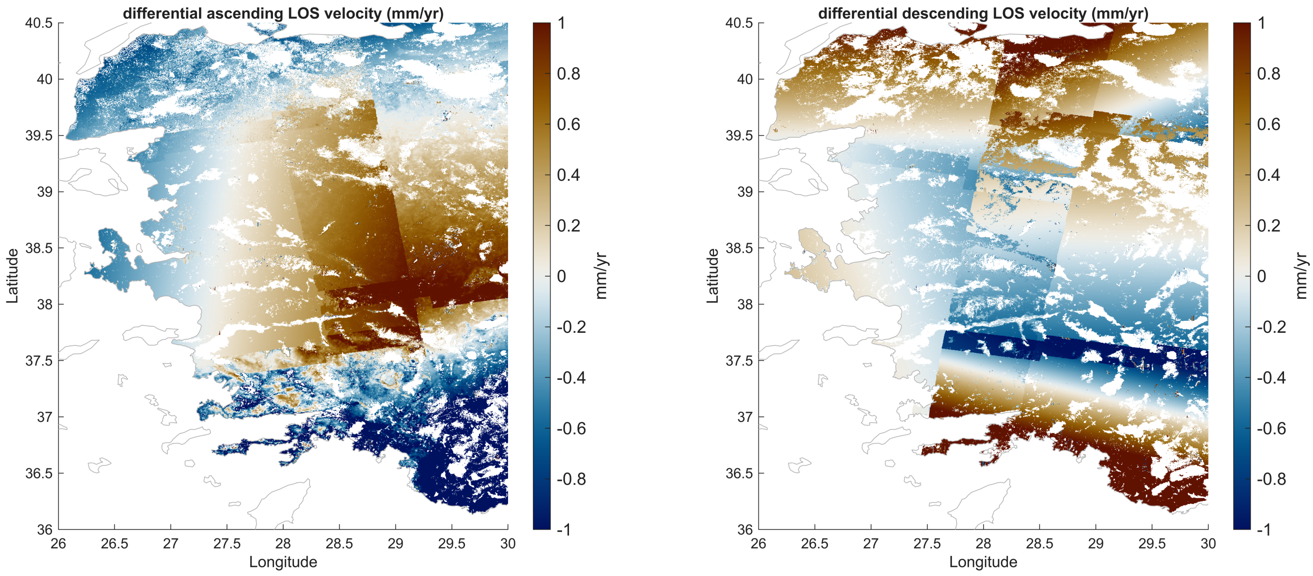

Figure S3. Comparison of InSAR velocities referenced to GNSS data from Özdemir & Karslıoğlu (2019), Nocquet et al. (2012), and England et al. (2016) with velocities referenced to newly released GNSS data by Kurt et al. (2023). Average differences are 0.0262 mm/yr for ascending and 0.1090 mm/yr for descending geometry, respectively.

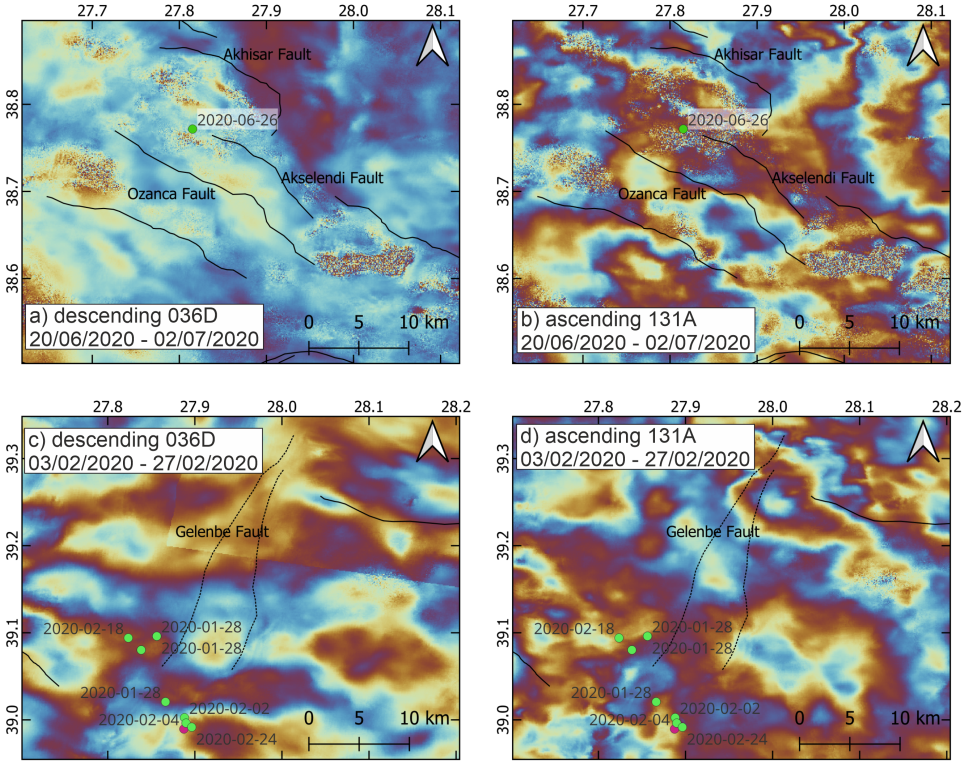

Figure S4. Interferograms covering earthquakes larger than M_W 5.0 showing no surface deformation. a and b show interferograms (descending and ascending frames) of the 26/06/2020 M_W 5.3 earthquake south of Akhisar (close to the Akselendi Fault. c and d show descending and ascending interferograms of the 04/02/2020 M_W 5.1 earthquake north of Akhisar, near the Gelenbe Fault, also covering three earthquakes with M_W 5.0 on 18th, 23rd, and 24th February 2020, though no clear deformation is visible on any nearby fault.

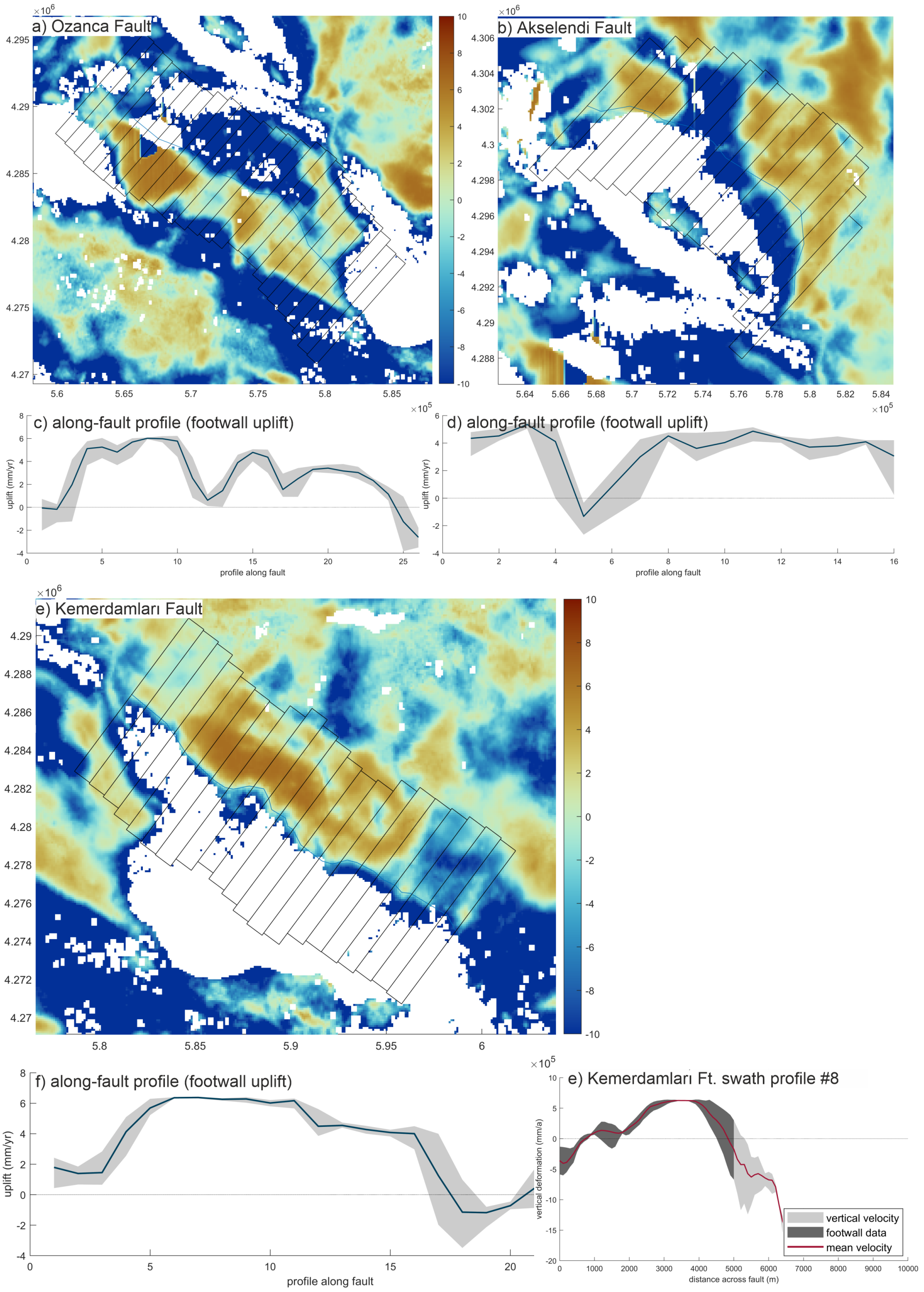

Figure S5. Detailed analyses of deformation along the (a) Ozanca, (b) Akselendi, and (e) Kemerdamları Faults in the western Gediz Graben. Figures c, d, and f show along-fault profiles of footwall uplift of the three faults, respectively, based on across-fault swath profiles. Figure g is an example of a swath profile showing uplift of up to ∼6 mm in the footwall. The hangingwall subsidence is clearly influenced by non-tectonic processes.

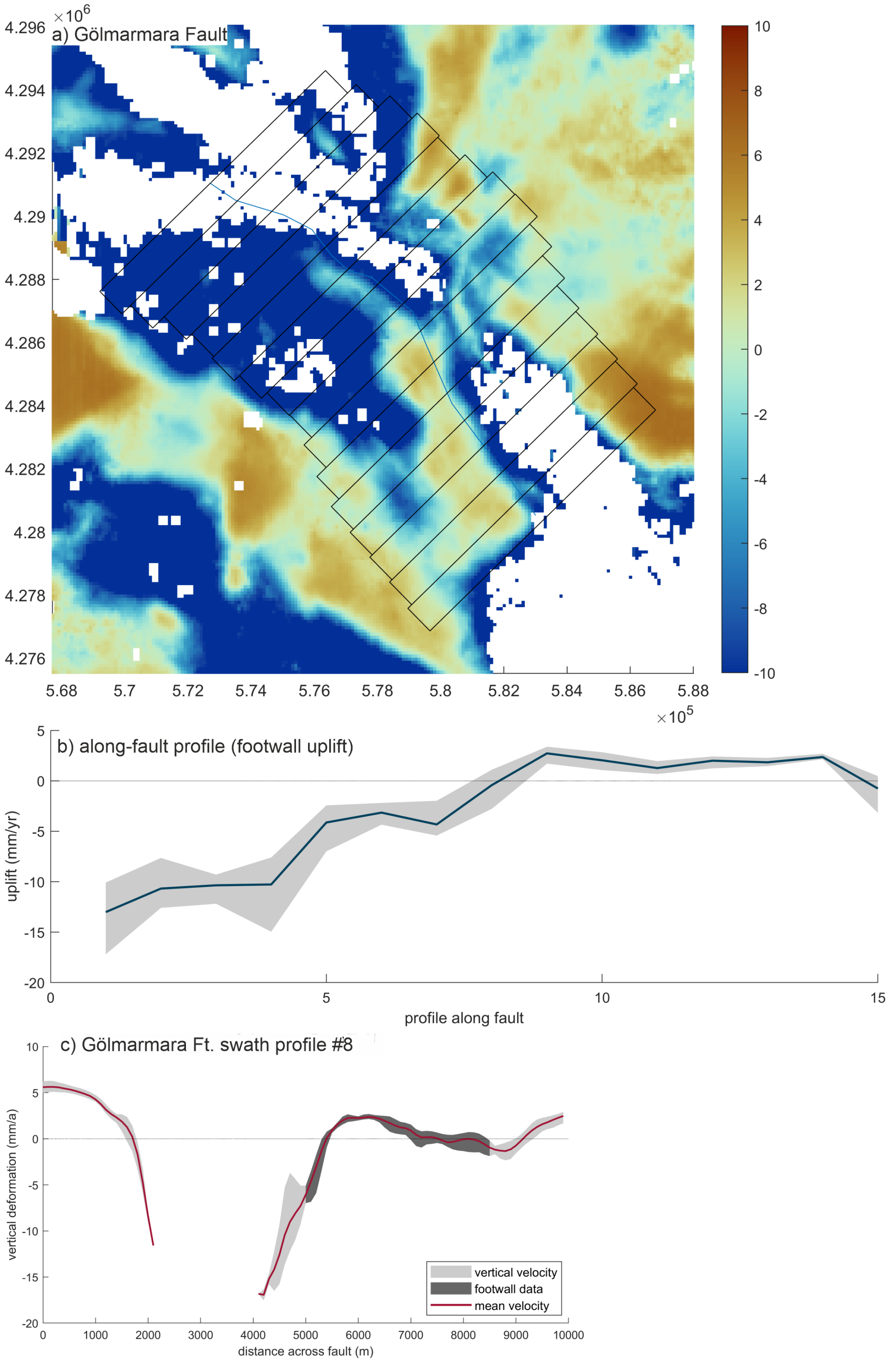

Figure S6. Detailed analyses of deformation along the (a) Gölmarmara Fault in the western Gediz Graben. Figure b shows the along-fault profile of footwall uplift based on across-fault swath profiles. Fig. c is an example of a swath profile showing the footwall uplift of the Gölmarmara Fault and subsidence in the hanging wall. The left side of the profile shows the uplift signal of the Kemerdamları Fault on the opposite graben margin.

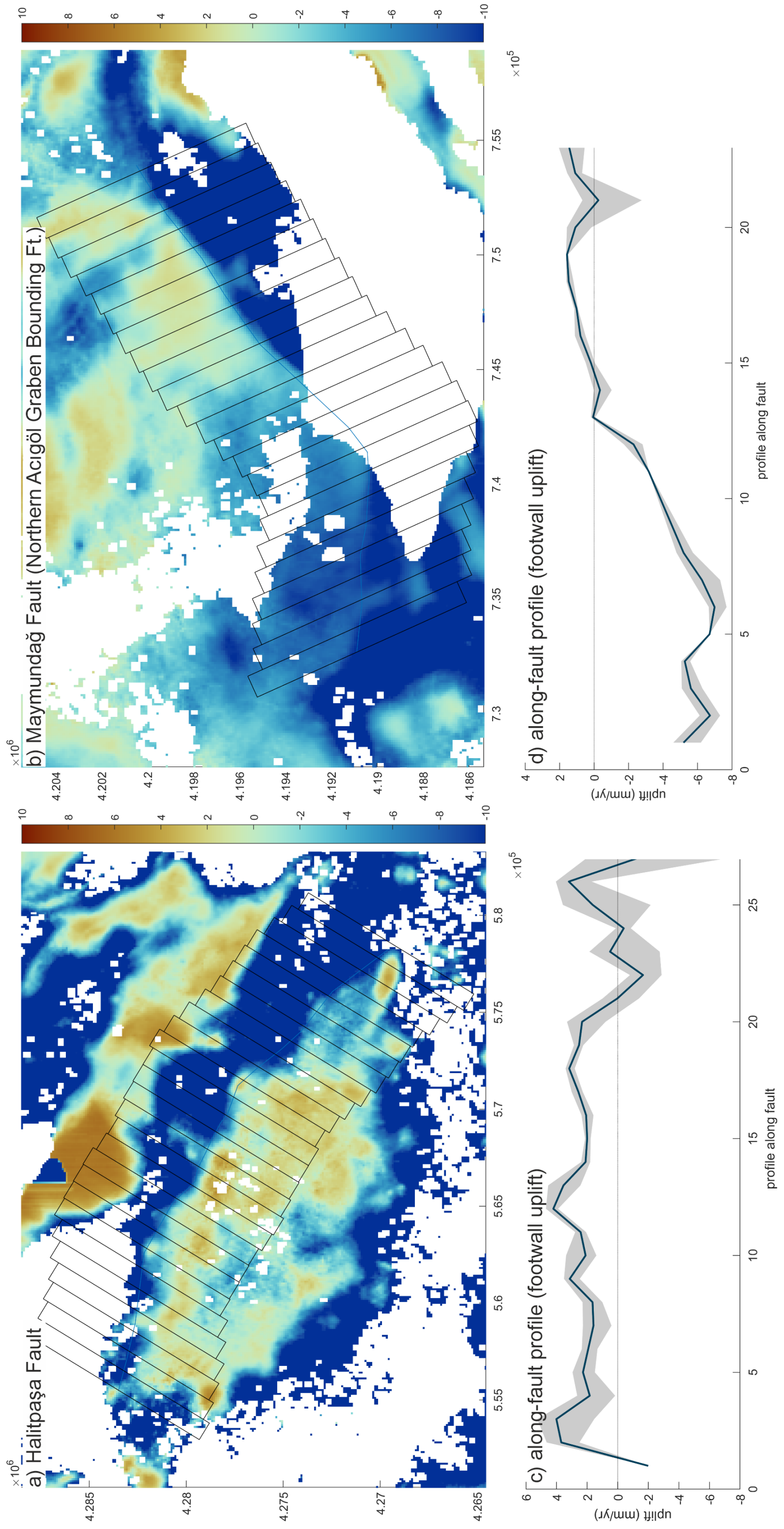

Figure S7. Detailed analyses of deformation along the (a) Halitpaşa Fault in the western Gediz Graben and the (b) Maymundağ Fault bounding the northern side of the Acıgöl Graben. Figures c, and d show along-fault profiles of footwall uplift based on across-fault swath profiles.

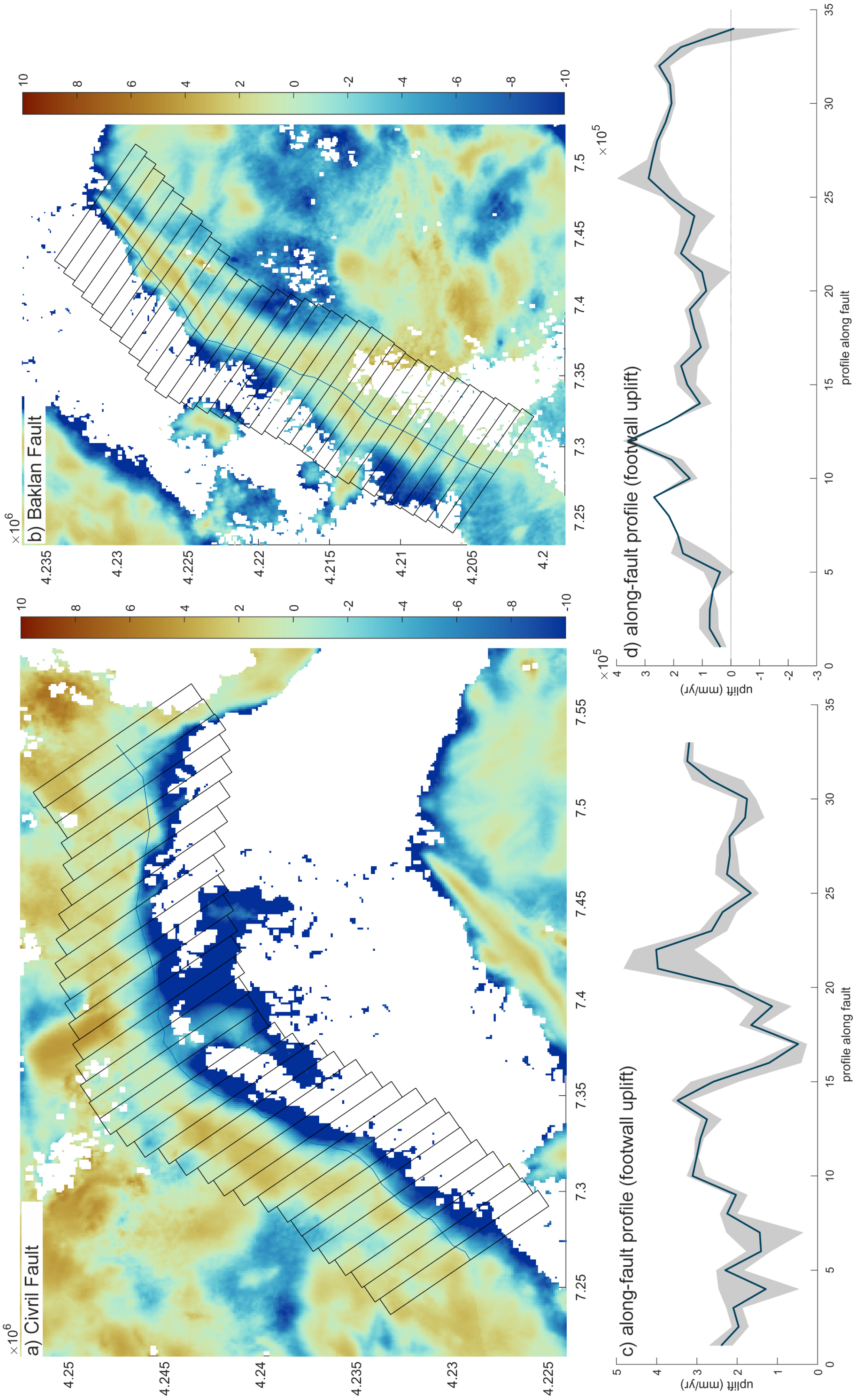

Figure S8. Detailed analyses of deformation along the (a) Çivril and (b) Baklan Faults bounding the Çivril Graben on its north-western and south-eastern sides, respectively. Figures c, and d show along-fault profiles of footwall uplift based on across-fault swath profiles.

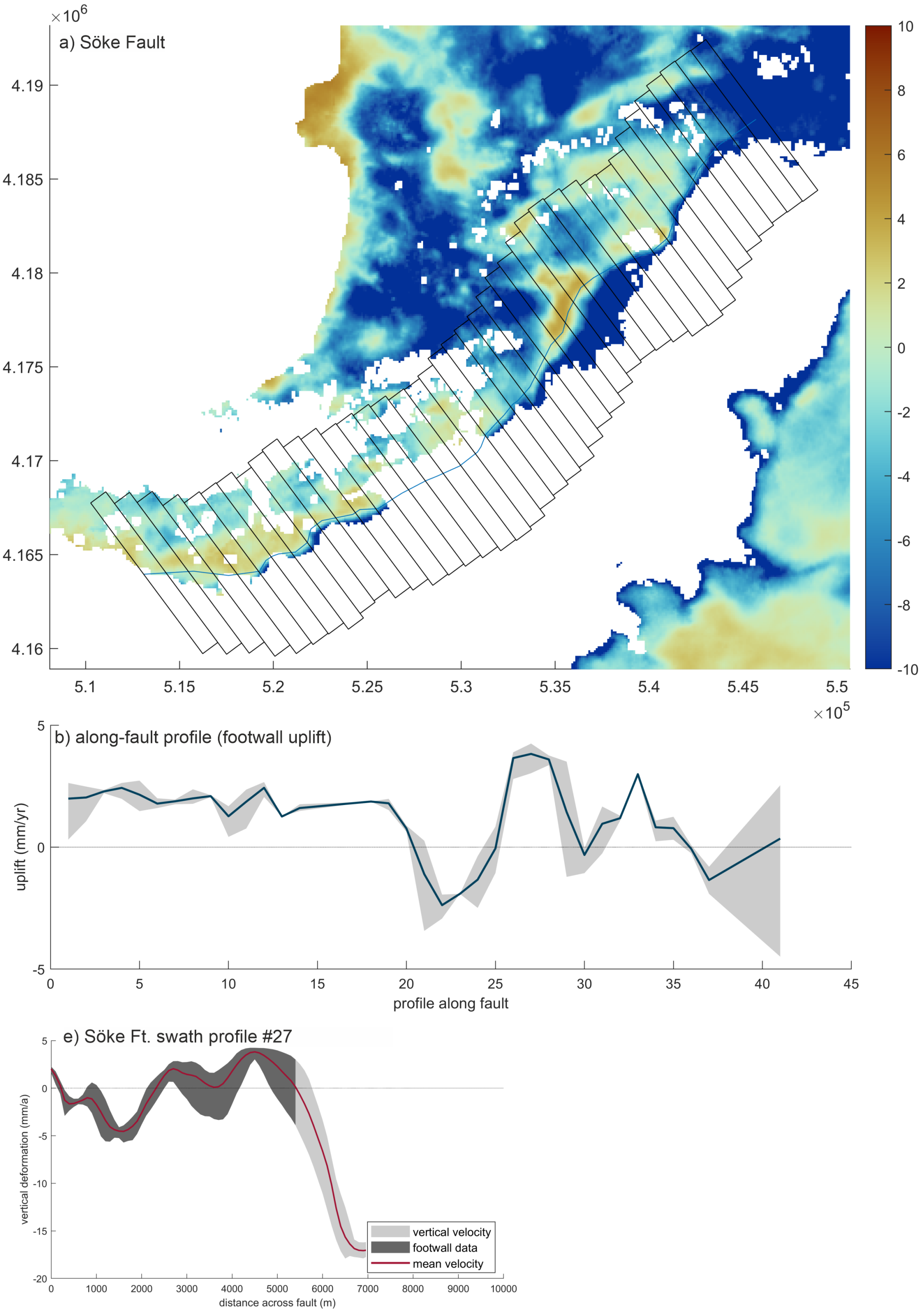

Figure S9. Detailed analyses of deformation along the Söke Fault.
