# Supplementary figures and images for: Active Deformation Across the Western Anatolian Extensional Province (Türkiye) From Sentinel‐1 InSAR
Source: Tectonics. 2024 Nov 24;43(11):e2023TC008086. doi: 10.1029/2023TC008086 (PMC11586515; doi:10.1029/2023TC008086)

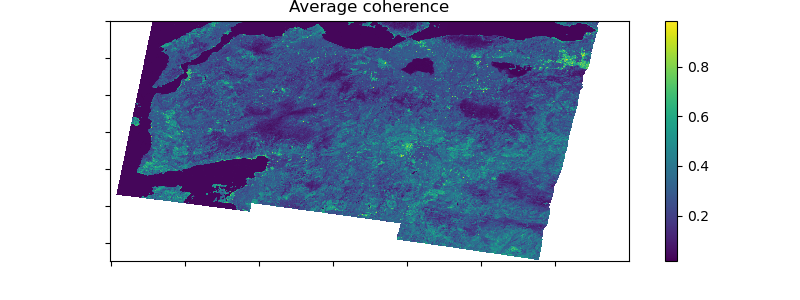

Supplement: Supplementary file 2 — Supporting Information S2 [file TECT-43-0-s001.zip › S3/036D_04976_TS/coh_avg.png]

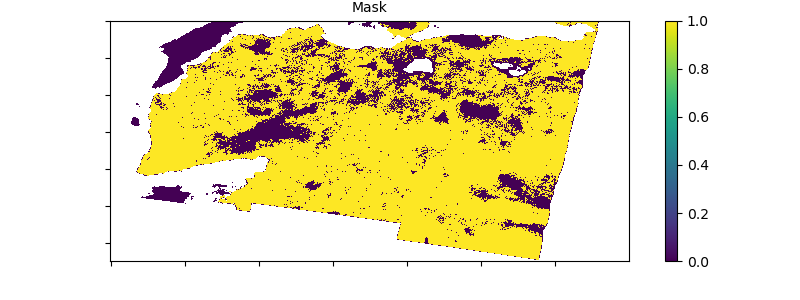

Supplement: Supplementary file 2 — Supporting Information S2 [file TECT-43-0-s001.zip › S3/036D_04976_TS/mask.png]

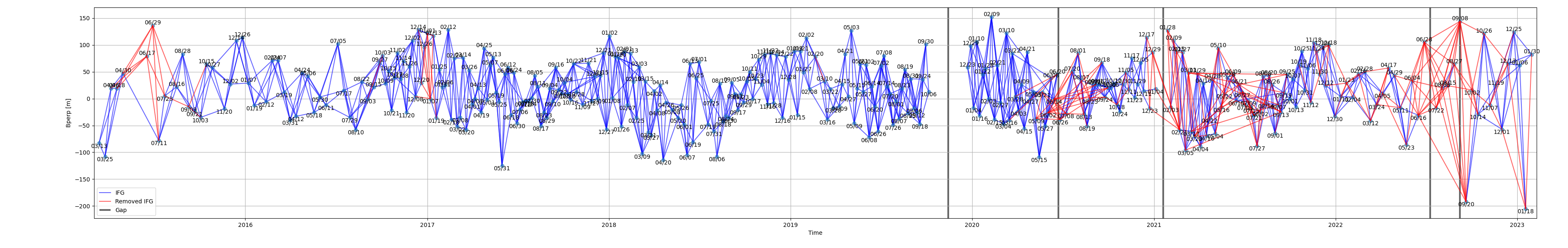

Supplement: Supplementary file 2 — Supporting Information S2 [file TECT-43-0-s001.zip › S3/036D_04976_TS/network13.png]

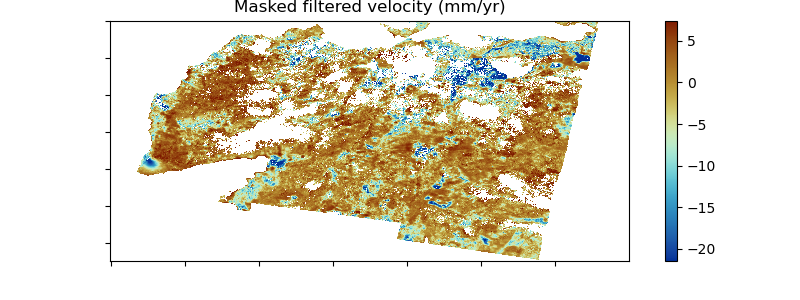

Supplement: Supplementary file 2 — Supporting Information S2 [file TECT-43-0-s001.zip › S3/036D_04976_TS/vel.filt.mskd.png]

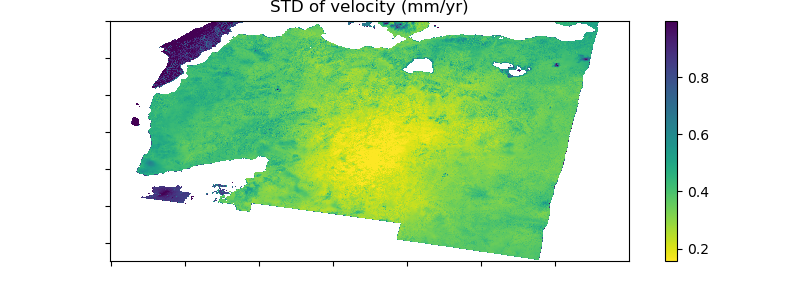

Supplement: Supplementary file 2 — Supporting Information S2 [file TECT-43-0-s001.zip › S3/036D_04976_TS/vstd.png]

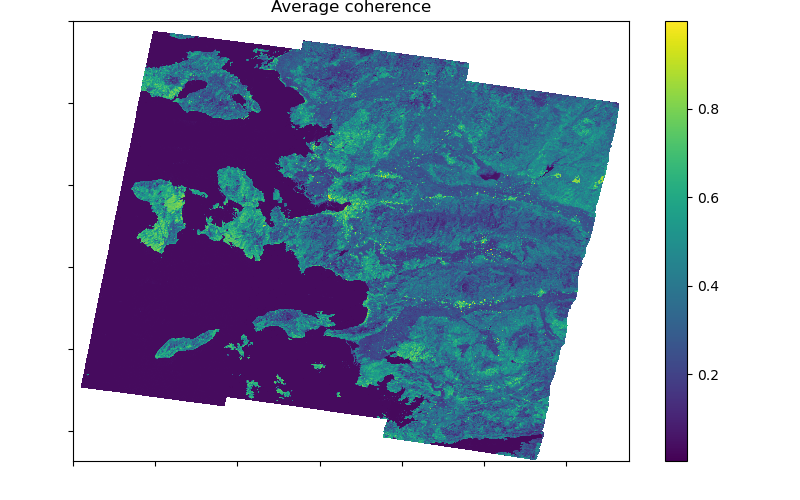

Supplement: Supplementary file 2 — Supporting Information S2 [file TECT-43-0-s001.zip › S3/036D_05175_TS/coh_avg.png]

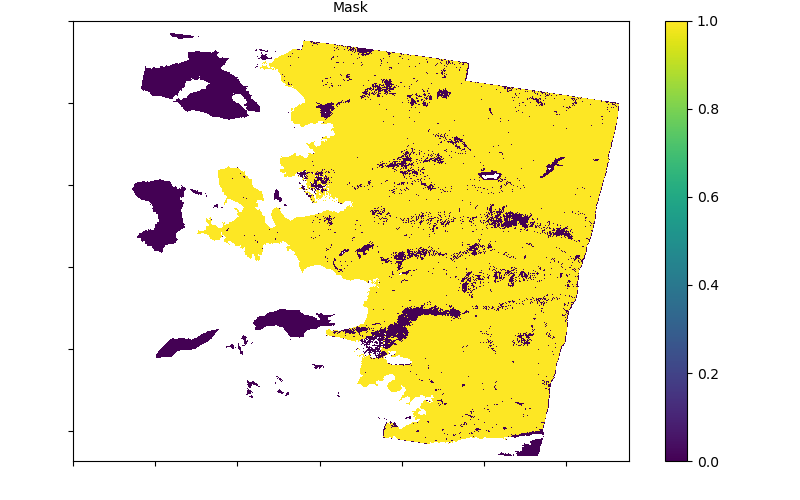

Supplement: Supplementary file 2 — Supporting Information S2 [file TECT-43-0-s001.zip › S3/036D_05175_TS/mask.png]

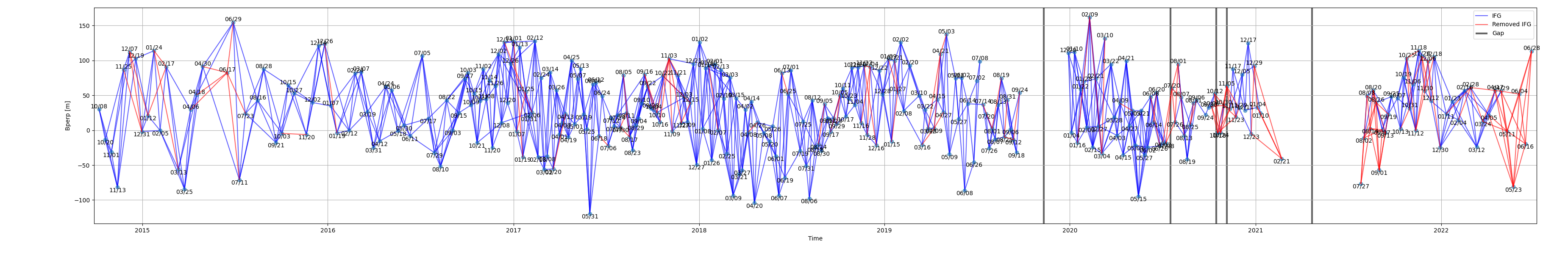

Supplement: Supplementary file 2 — Supporting Information S2 [file TECT-43-0-s001.zip › S3/036D_05175_TS/network13.png]

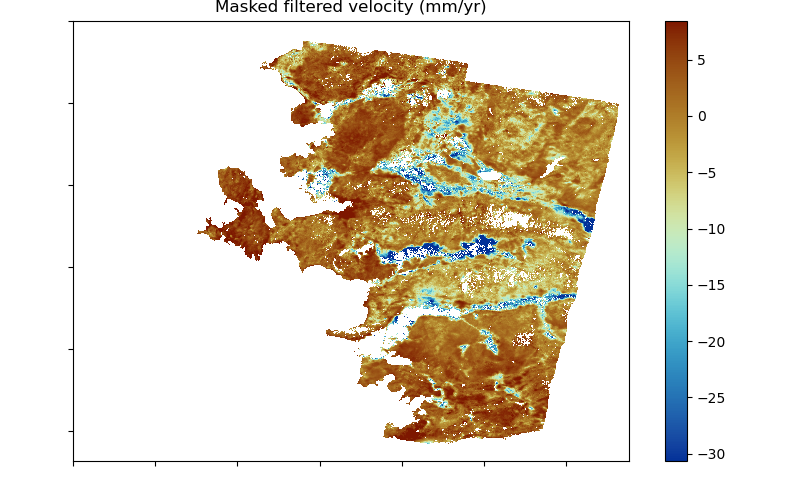

Supplement: Supplementary file 2 — Supporting Information S2 [file TECT-43-0-s001.zip › S3/036D_05175_TS/vel.filt.mskd.png]

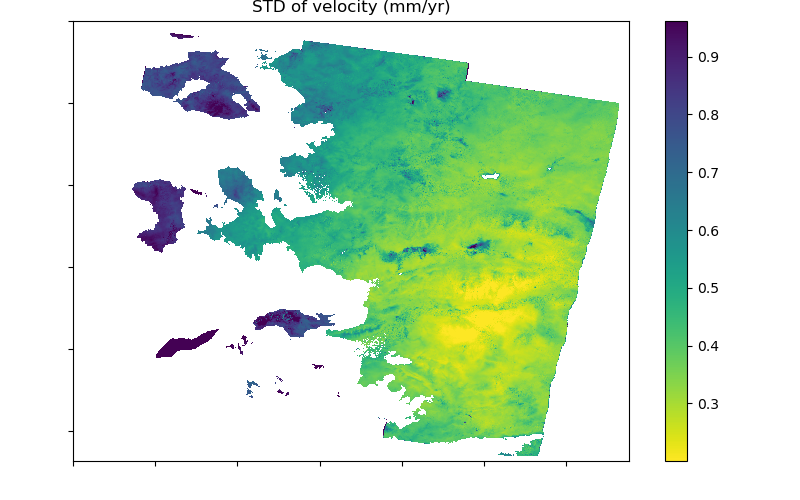

Supplement: Supplementary file 2 — Supporting Information S2 [file TECT-43-0-s001.zip › S3/036D_05175_TS/vstd.png]

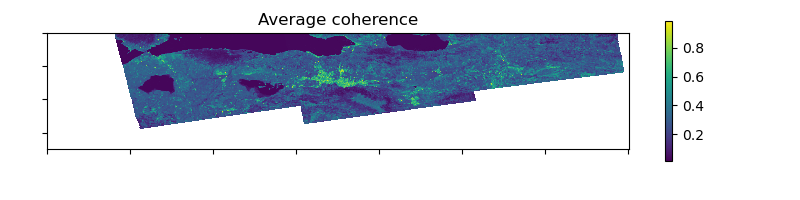

Supplement: Supplementary file 2 — Supporting Information S2 [file TECT-43-0-s001.zip › S3/058A_04914_TS/coh_avg.png]

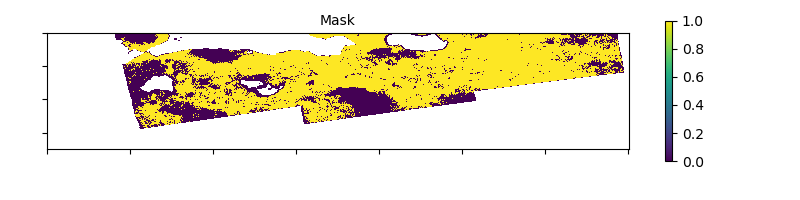

Supplement: Supplementary file 2 — Supporting Information S2 [file TECT-43-0-s001.zip › S3/058A_04914_TS/mask.png]

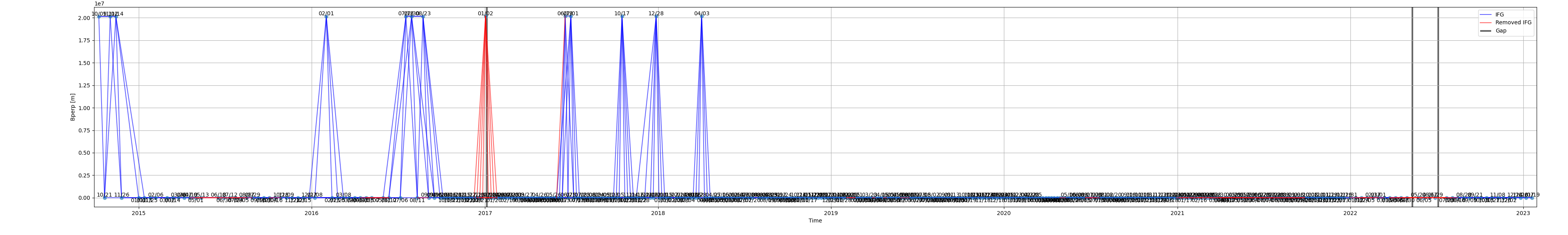

Supplement: Supplementary file 2 — Supporting Information S2 [file TECT-43-0-s001.zip › S3/058A_04914_TS/network13.png]

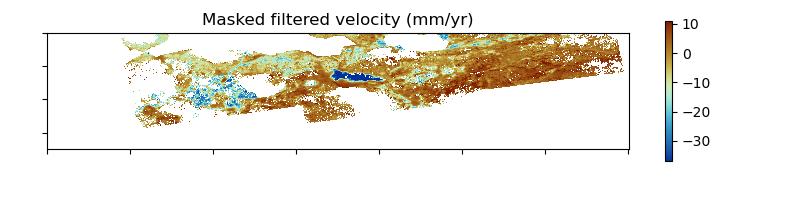

Supplement: Supplementary file 2 — Supporting Information S2 [file TECT-43-0-s001.zip › S3/058A_04914_TS/vel.filt.mskd.png]

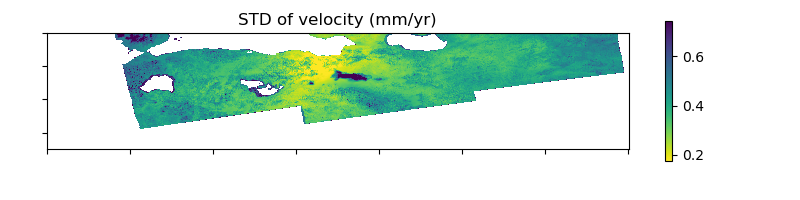

Supplement: Supplementary file 2 — Supporting Information S2 [file TECT-43-0-s001.zip › S3/058A_04914_TS/vstd.png]

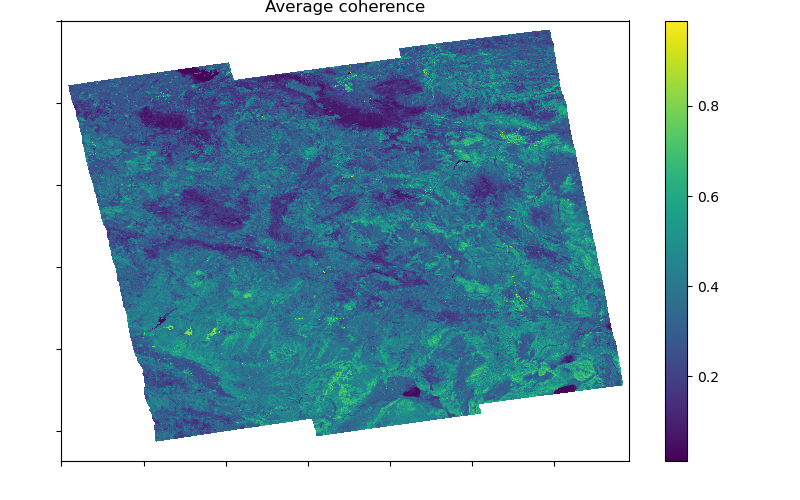

Supplement: Supplementary file 2 — Supporting Information S2 [file TECT-43-0-s001.zip › S3/058A_05086_TS/coh_avg.png]

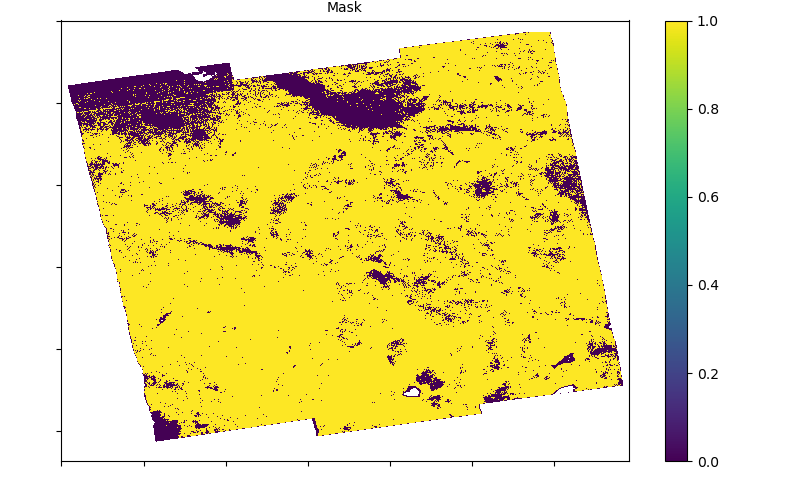

Supplement: Supplementary file 2 — Supporting Information S2 [file TECT-43-0-s001.zip › S3/058A_05086_TS/mask.png]

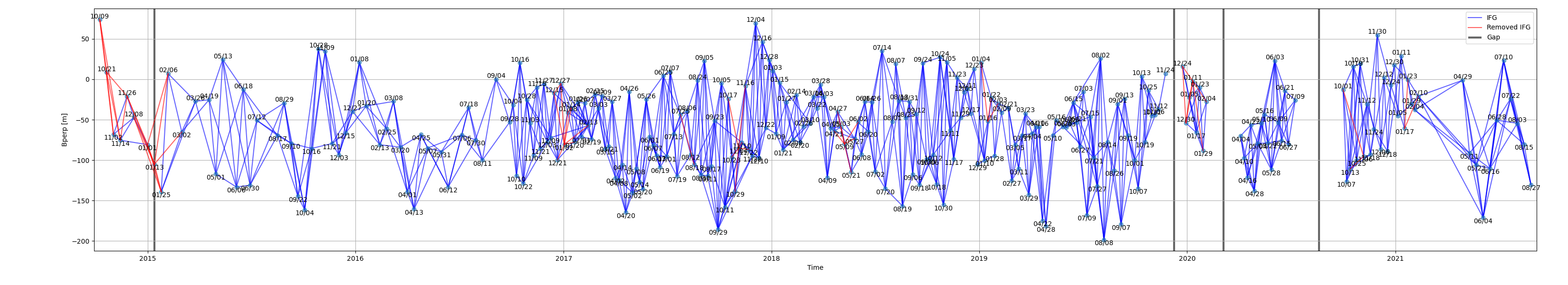

Supplement: Supplementary file 2 — Supporting Information S2 [file TECT-43-0-s001.zip › S3/058A_05086_TS/network13.png]

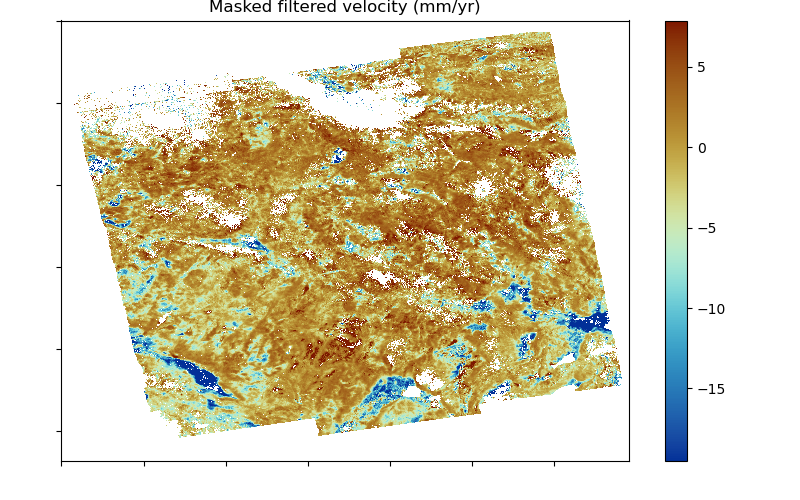

Supplement: Supplementary file 2 — Supporting Information S2 [file TECT-43-0-s001.zip › S3/058A_05086_TS/vel.filt.mskd.png]

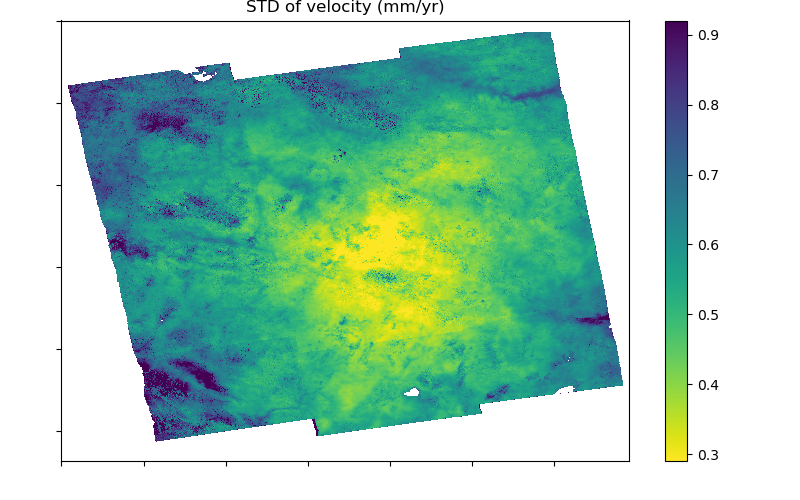

Supplement: Supplementary file 2 — Supporting Information S2 [file TECT-43-0-s001.zip › S3/058A_05086_TS/vstd.png]

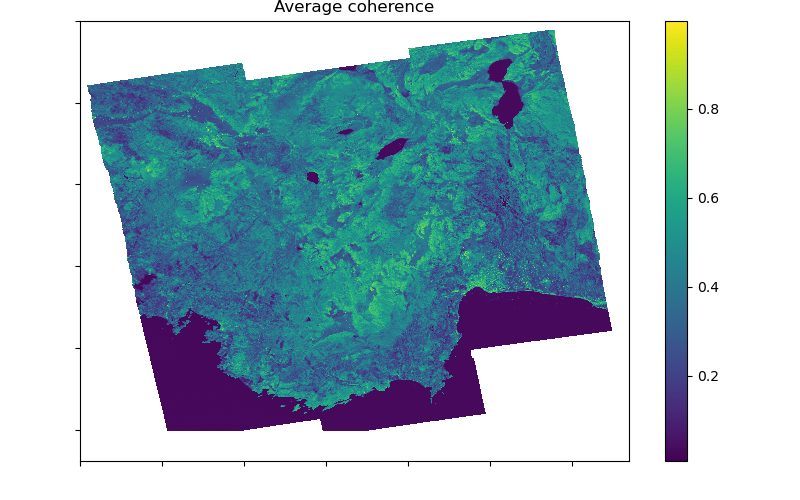

Supplement: Supplementary file 2 — Supporting Information S2 [file TECT-43-0-s001.zip › S3/058A_05279_TS/coh_avg.png]

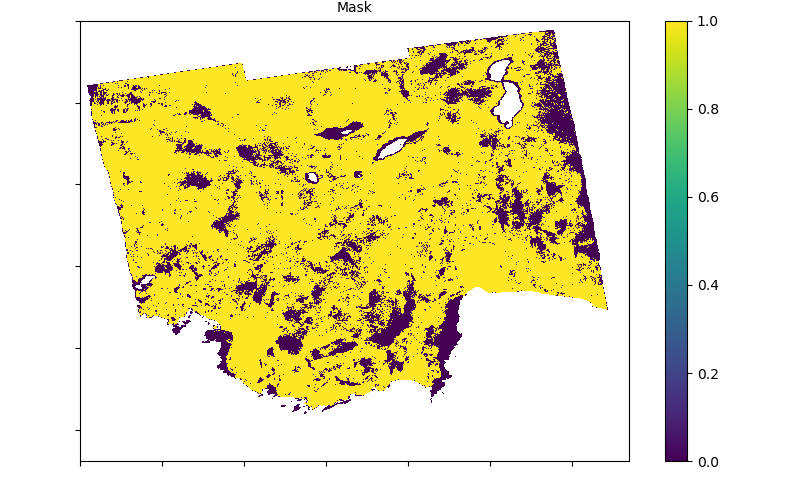

Supplement: Supplementary file 2 — Supporting Information S2 [file TECT-43-0-s001.zip › S3/058A_05279_TS/mask.png]

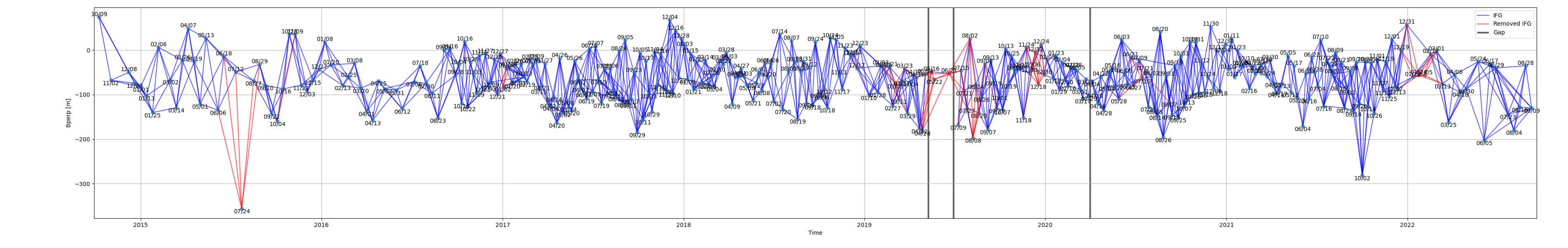

Supplement: Supplementary file 2 — Supporting Information S2 [file TECT-43-0-s001.zip › S3/058A_05279_TS/network13.png]

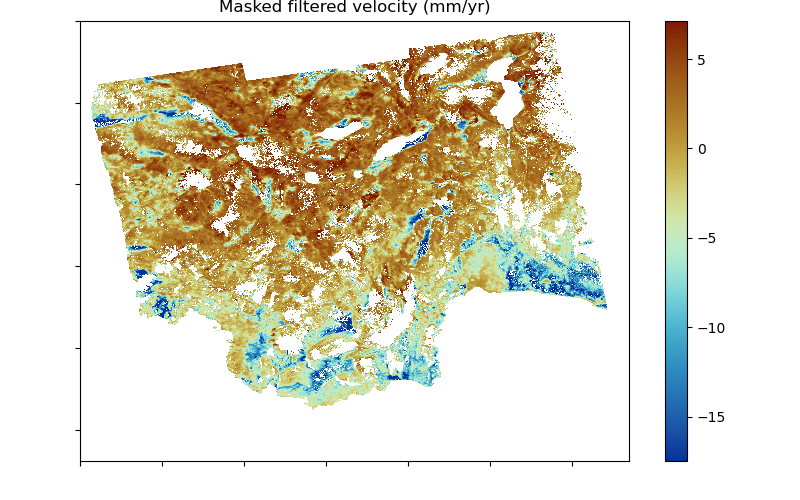

Supplement: Supplementary file 2 — Supporting Information S2 [file TECT-43-0-s001.zip › S3/058A_05279_TS/vel.filt.mskd.png]

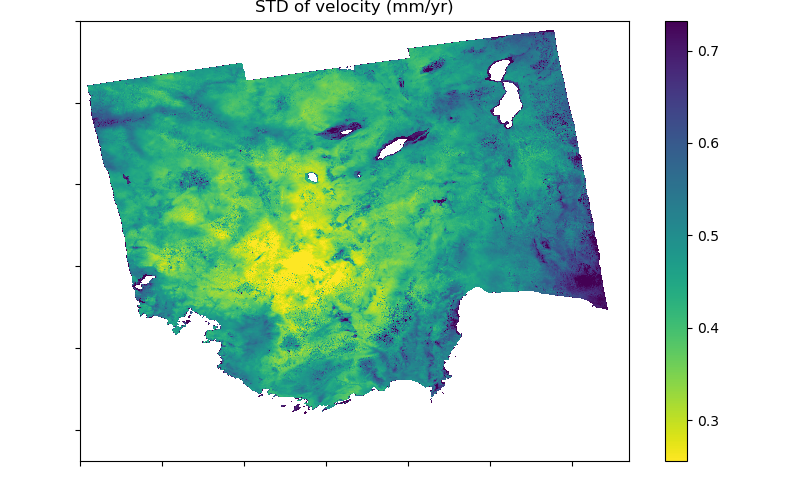

Supplement: Supplementary file 2 — Supporting Information S2 [file TECT-43-0-s001.zip › S3/058A_05279_TS/vstd.png]

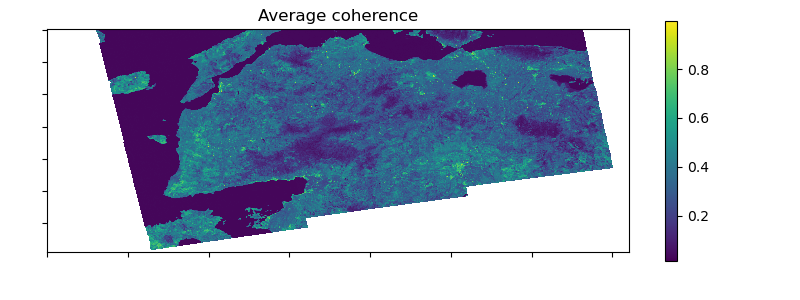

Supplement: Supplementary file 2 — Supporting Information S2 [file TECT-43-0-s001.zip › S3/131A_04951_TS/coh_avg.png]

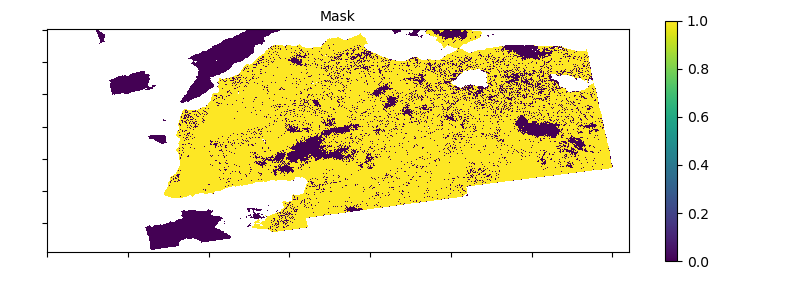

Supplement: Supplementary file 2 — Supporting Information S2 [file TECT-43-0-s001.zip › S3/131A_04951_TS/mask.png]

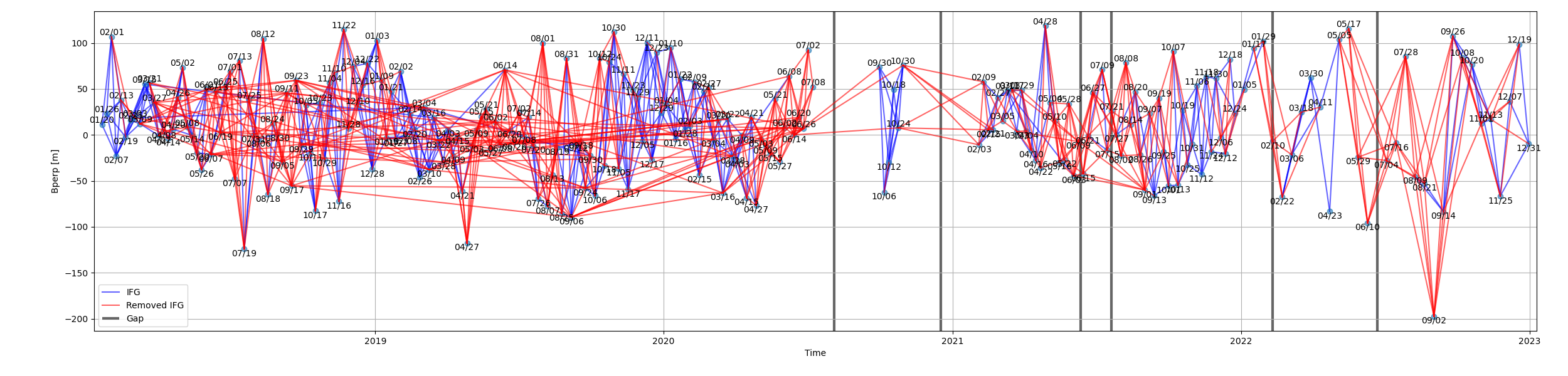

Supplement: Supplementary file 2 — Supporting Information S2 [file TECT-43-0-s001.zip › S3/131A_04951_TS/network13.png]

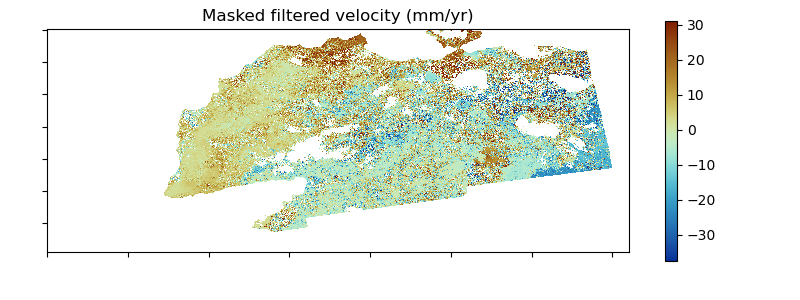

Supplement: Supplementary file 2 — Supporting Information S2 [file TECT-43-0-s001.zip › S3/131A_04951_TS/vel.filt.mskd.png]

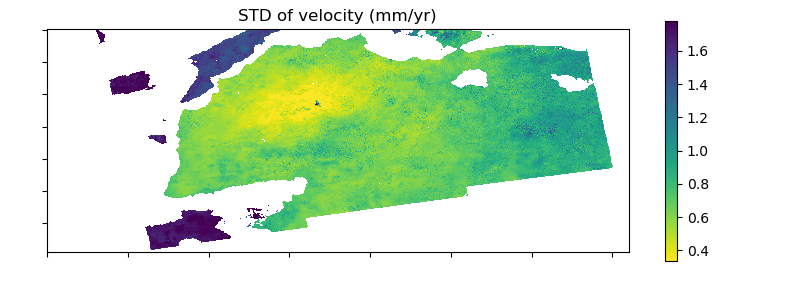

Supplement: Supplementary file 2 — Supporting Information S2 [file TECT-43-0-s001.zip › S3/131A_04951_TS/vstd.png]

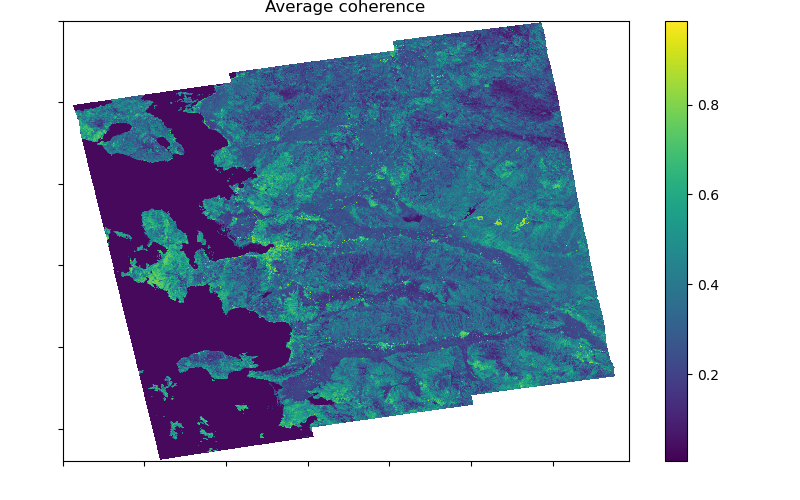

Supplement: Supplementary file 2 — Supporting Information S2 [file TECT-43-0-s001.zip › S3/131A_05153_TS/coh_avg.png]

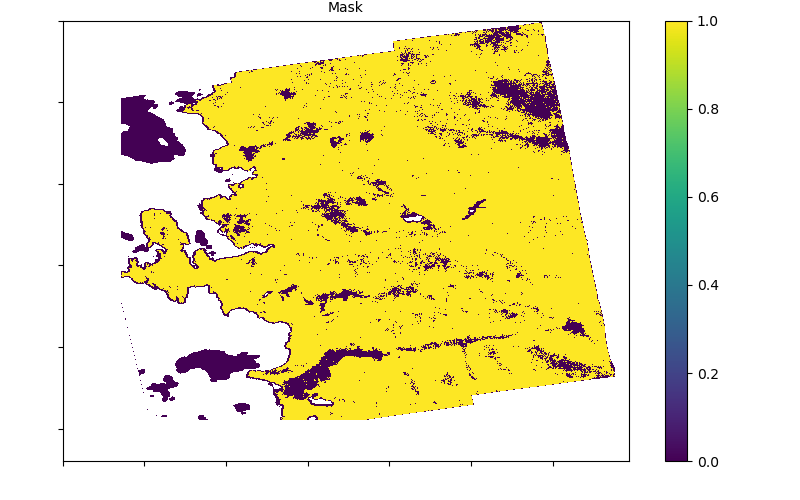

Supplement: Supplementary file 2 — Supporting Information S2 [file TECT-43-0-s001.zip › S3/131A_05153_TS/mask.png]

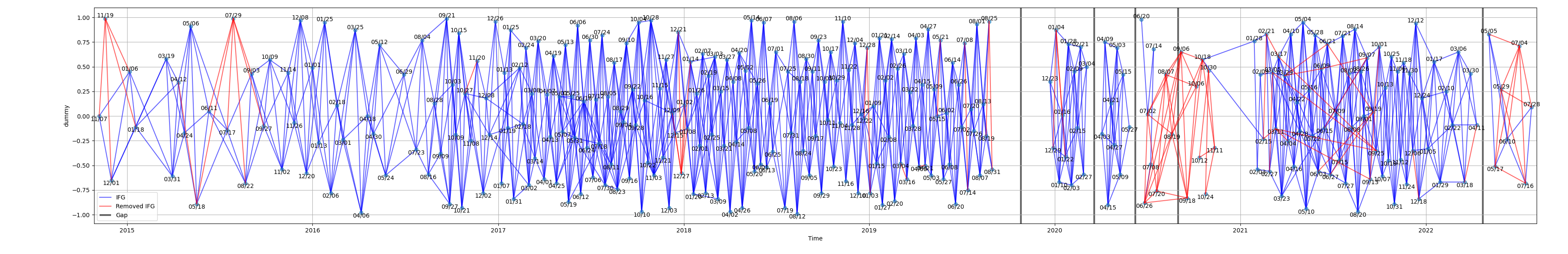

Supplement: Supplementary file 2 — Supporting Information S2 [file TECT-43-0-s001.zip › S3/131A_05153_TS/network13.png]

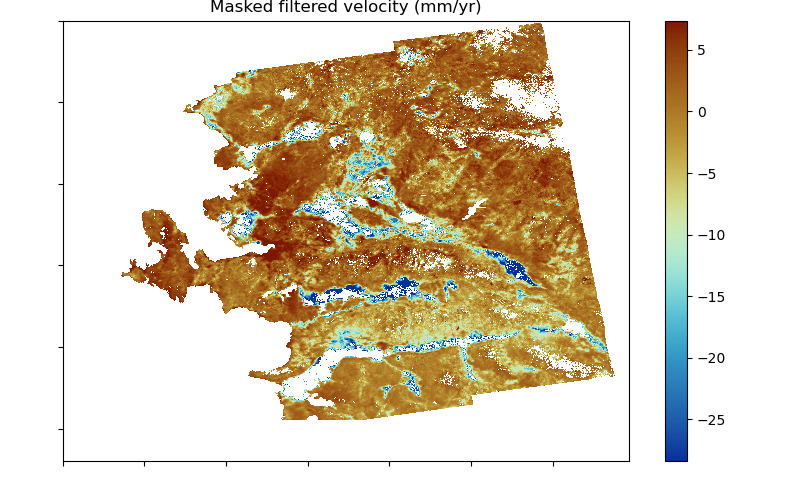

Supplement: Supplementary file 2 — Supporting Information S2 [file TECT-43-0-s001.zip › S3/131A_05153_TS/vel.filt.mskd.png]

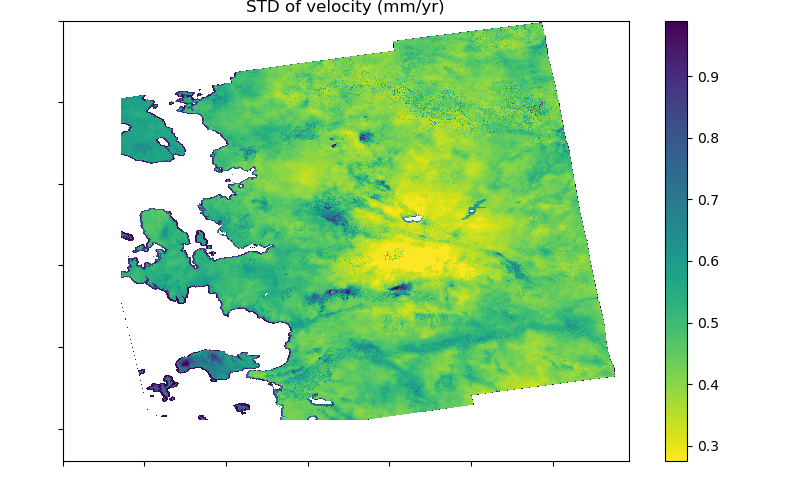

Supplement: Supplementary file 2 — Supporting Information S2 [file TECT-43-0-s001.zip › S3/131A_05153_TS/vstd.png]

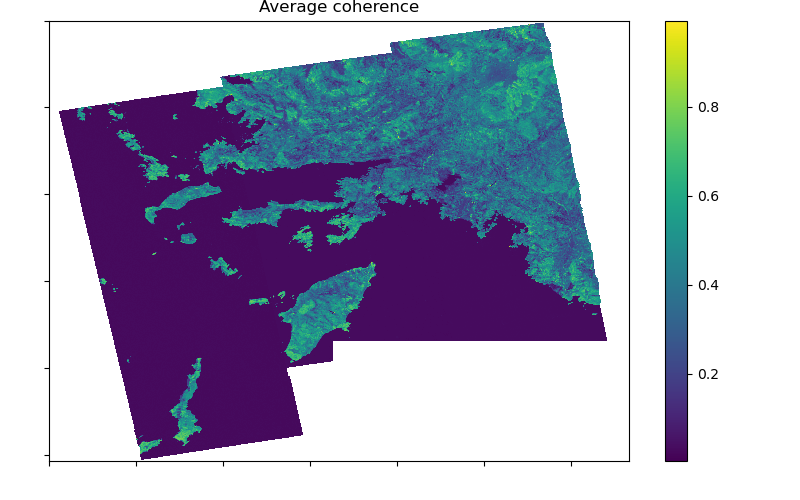

Supplement: Supplementary file 2 — Supporting Information S2 [file TECT-43-0-s001.zip › S3/131A_05336_TS/coh_avg.png]

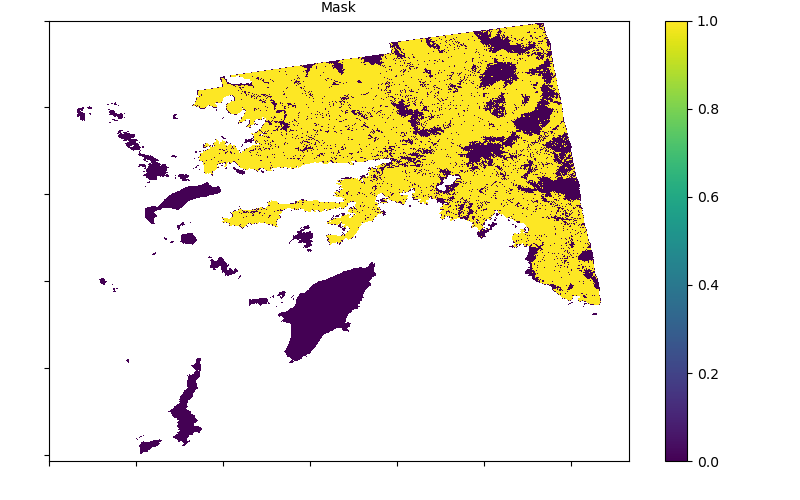

Supplement: Supplementary file 2 — Supporting Information S2 [file TECT-43-0-s001.zip › S3/131A_05336_TS/mask.png]

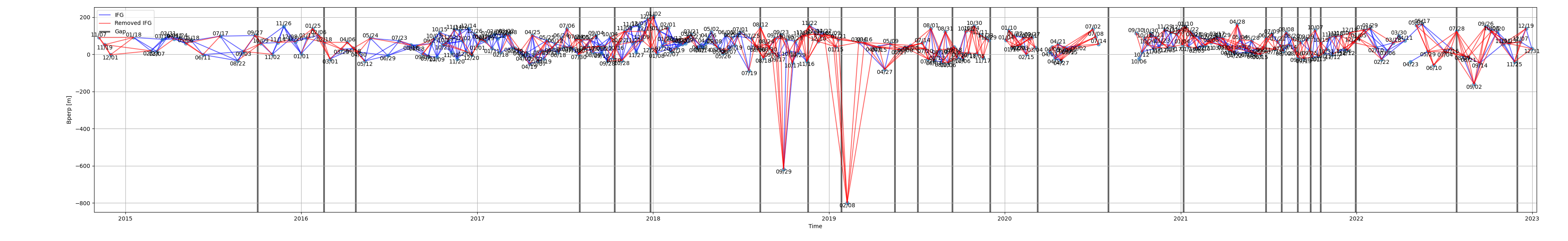

Supplement: Supplementary file 2 — Supporting Information S2 [file TECT-43-0-s001.zip › S3/131A_05336_TS/network13.png]

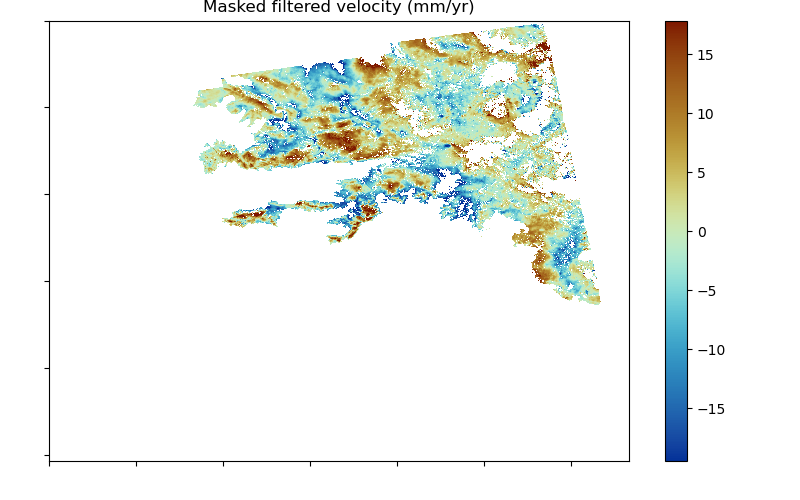

Supplement: Supplementary file 2 — Supporting Information S2 [file TECT-43-0-s001.zip › S3/131A_05336_TS/vel.filt.mskd.png]

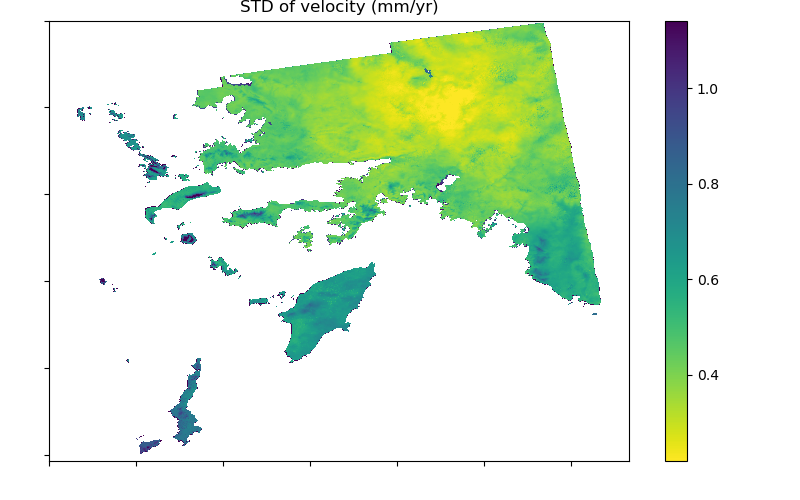

Supplement: Supplementary file 2 — Supporting Information S2 [file TECT-43-0-s001.zip › S3/131A_05336_TS/vstd.png]

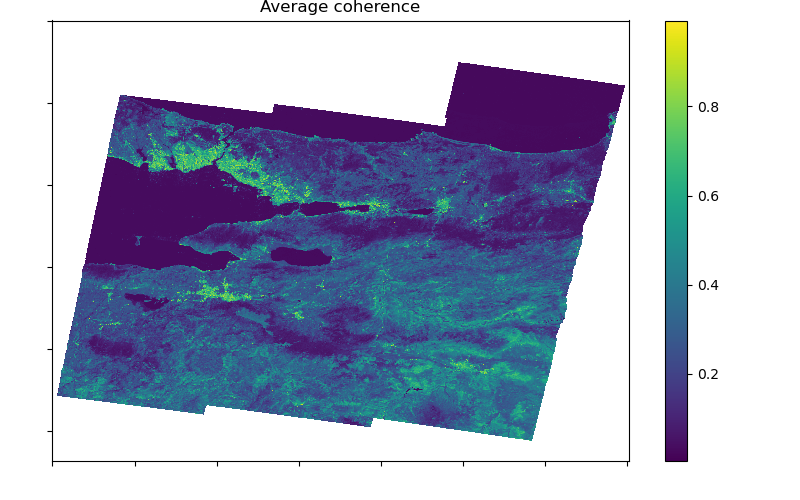

Supplement: Supplementary file 2 — Supporting Information S2 [file TECT-43-0-s001.zip › S3/138D_04954_TS/coh_avg.png]

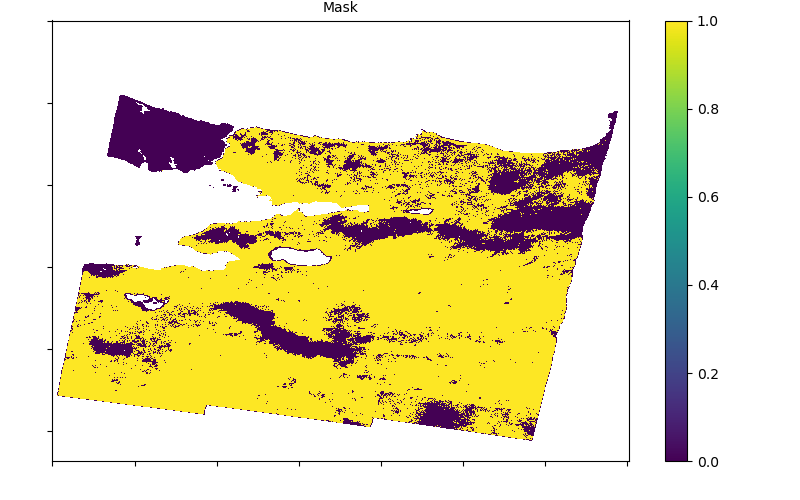

Supplement: Supplementary file 2 — Supporting Information S2 [file TECT-43-0-s001.zip › S3/138D_04954_TS/mask.png]

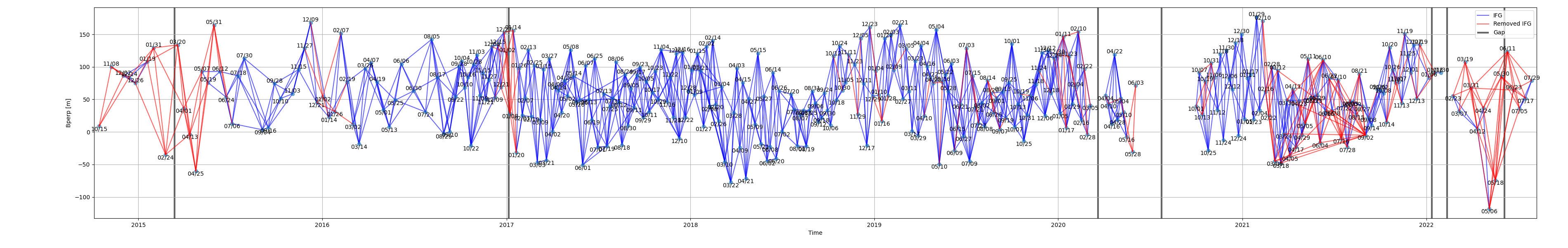

Supplement: Supplementary file 2 — Supporting Information S2 [file TECT-43-0-s001.zip › S3/138D_04954_TS/network13.png]

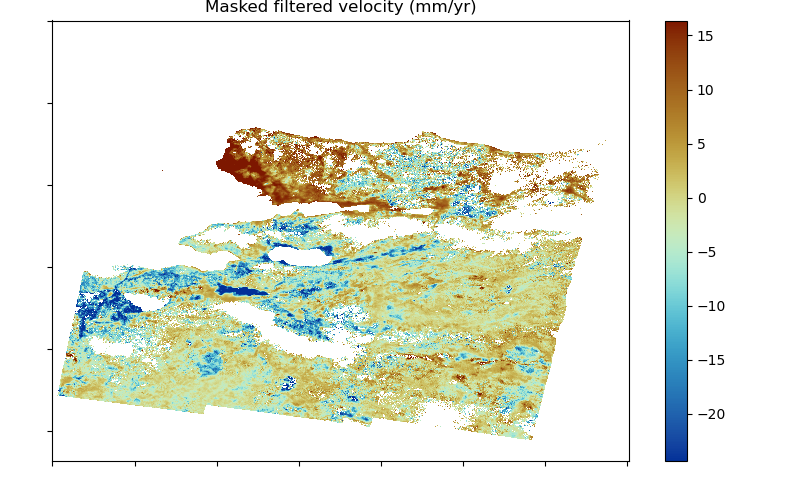

Supplement: Supplementary file 2 — Supporting Information S2 [file TECT-43-0-s001.zip › S3/138D_04954_TS/vel.filt.mskd.png]

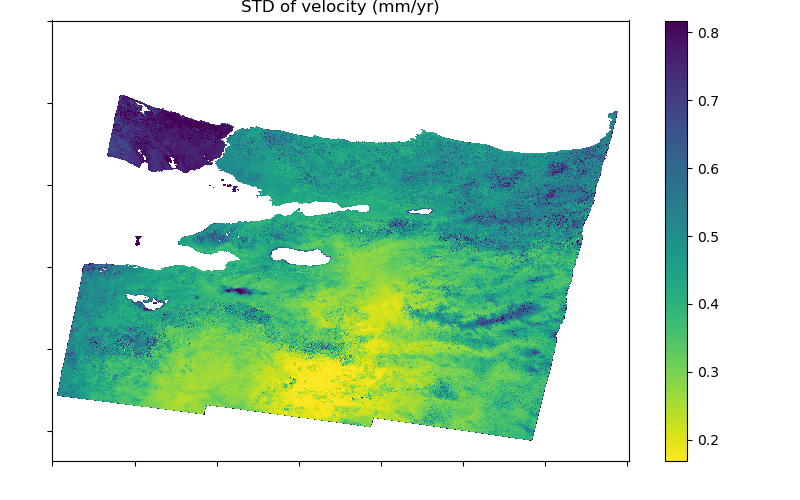

Supplement: Supplementary file 2 — Supporting Information S2 [file TECT-43-0-s001.zip › S3/138D_04954_TS/vstd.png]

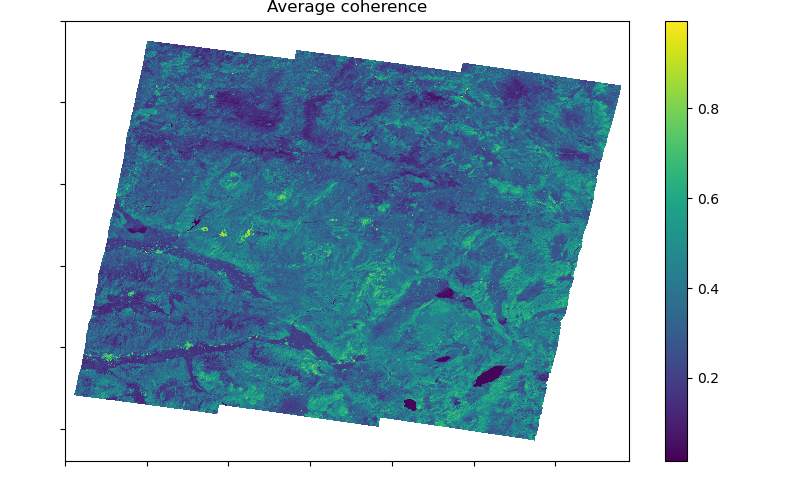

Supplement: Supplementary file 2 — Supporting Information S2 [file TECT-43-0-s001.zip › S3/138D_05142_TS/coh_avg.png]

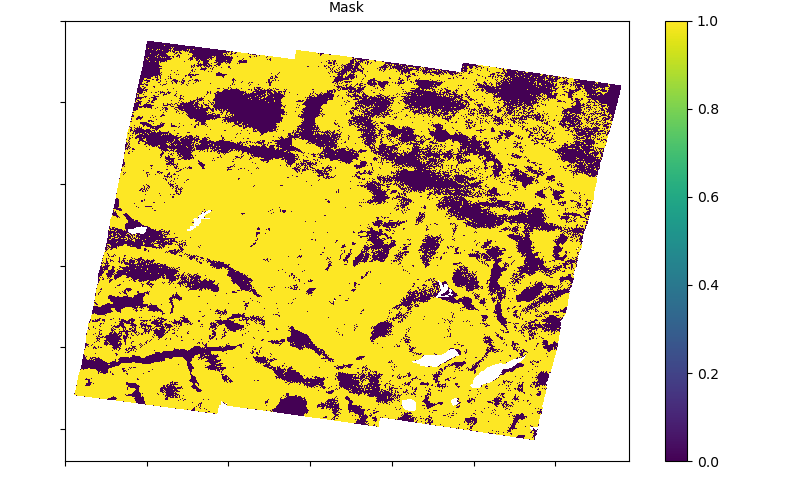

Supplement: Supplementary file 2 — Supporting Information S2 [file TECT-43-0-s001.zip › S3/138D_05142_TS/mask.png]

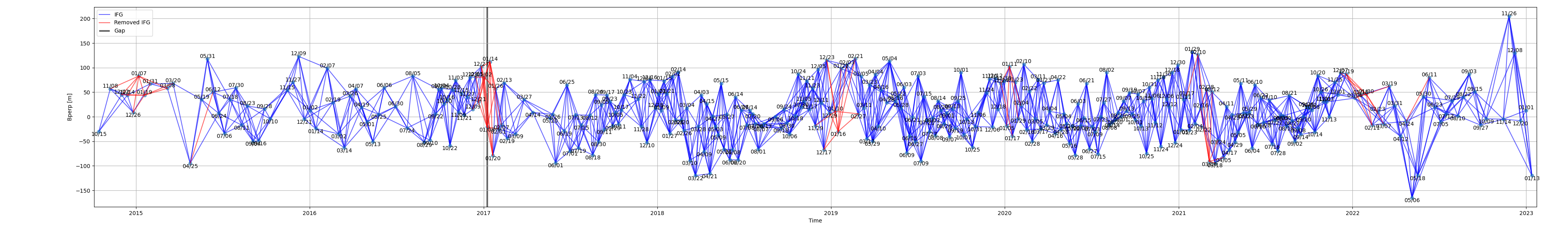

Supplement: Supplementary file 2 — Supporting Information S2 [file TECT-43-0-s001.zip › S3/138D_05142_TS/network13.png]

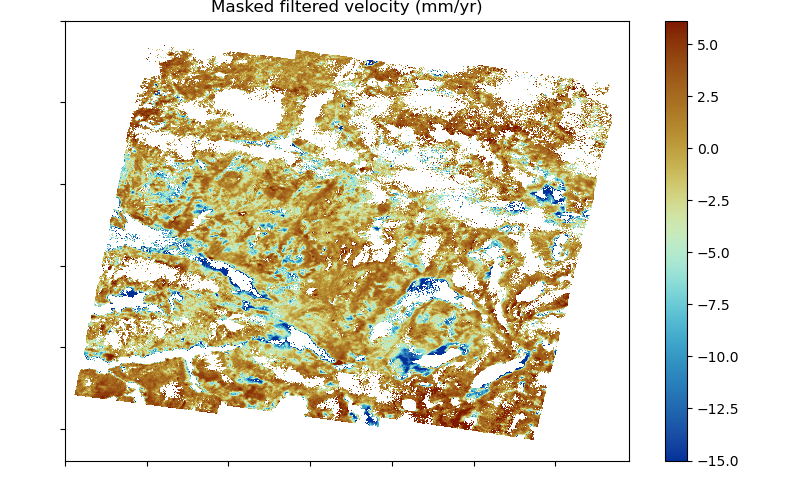

Supplement: Supplementary file 2 — Supporting Information S2 [file TECT-43-0-s001.zip › S3/138D_05142_TS/vel.filt.mskd.png]

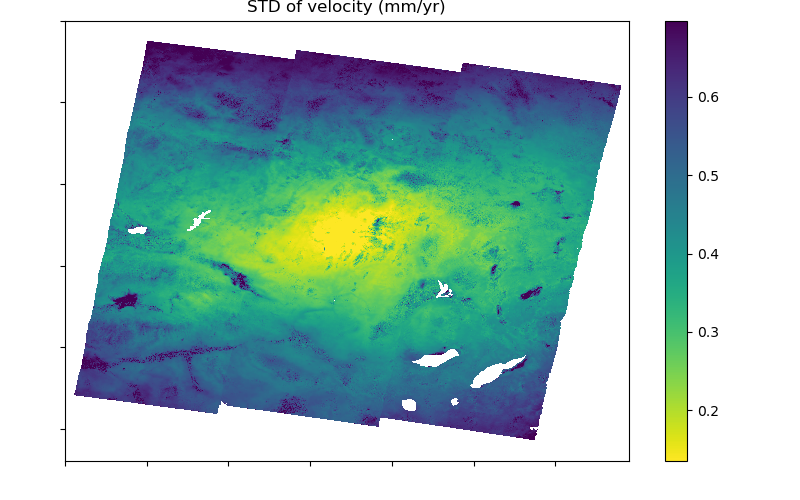

Supplement: Supplementary file 2 — Supporting Information S2 [file TECT-43-0-s001.zip › S3/138D_05142_TS/vstd.png]

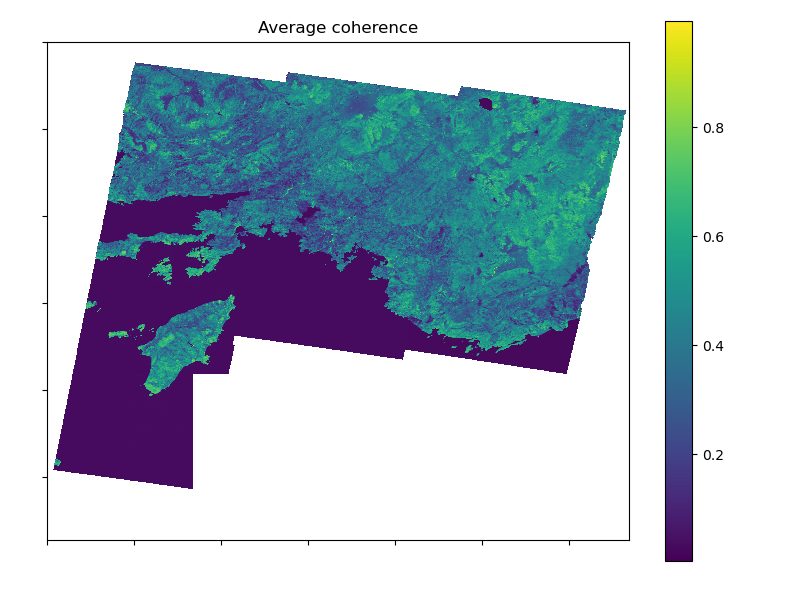

Supplement: Supplementary file 2 — Supporting Information S2 [file TECT-43-0-s001.zip › S3/138D_05325_TS/coh_avg.png]

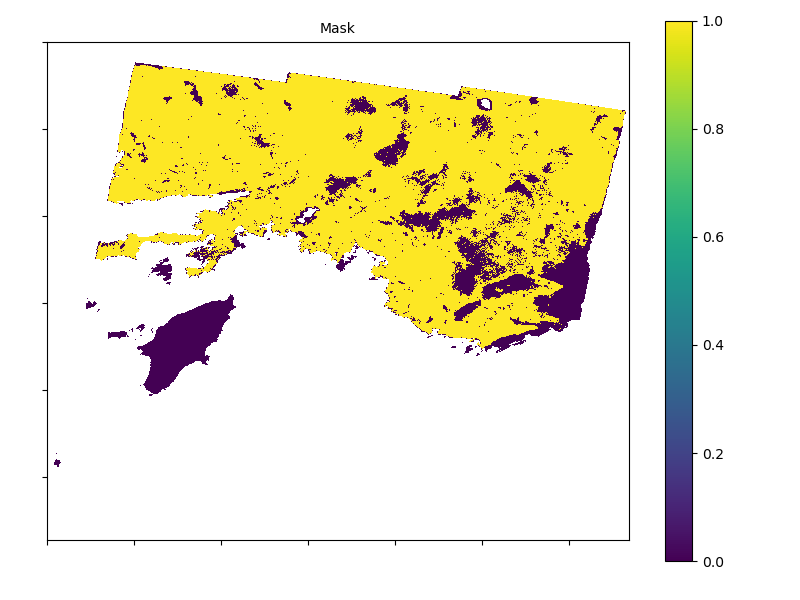

Supplement: Supplementary file 2 — Supporting Information S2 [file TECT-43-0-s001.zip › S3/138D_05325_TS/mask.png]

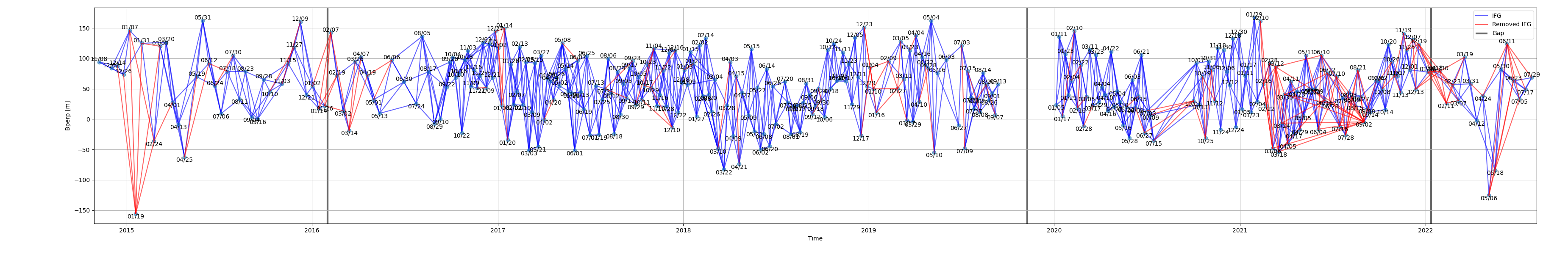

Supplement: Supplementary file 2 — Supporting Information S2 [file TECT-43-0-s001.zip › S3/138D_05325_TS/network13.png]

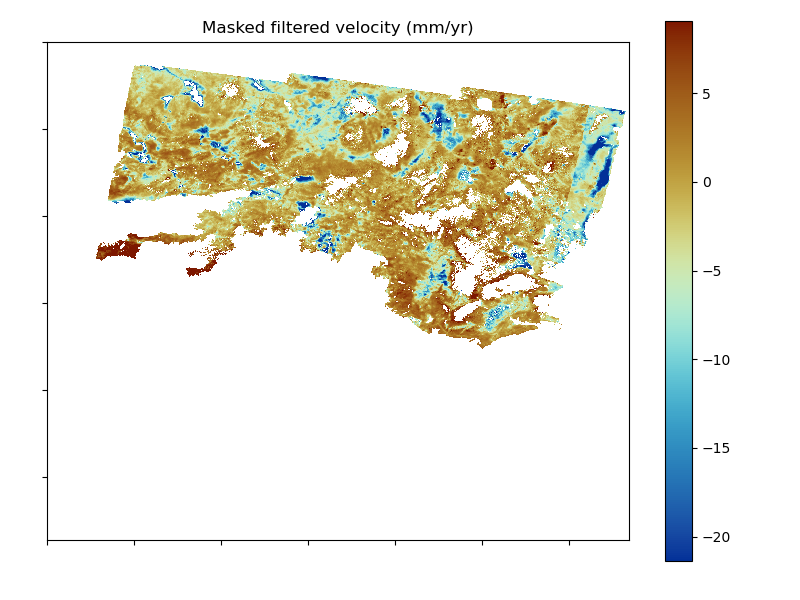

Supplement: Supplementary file 2 — Supporting Information S2 [file TECT-43-0-s001.zip › S3/138D_05325_TS/vel.filt.mskd.png]

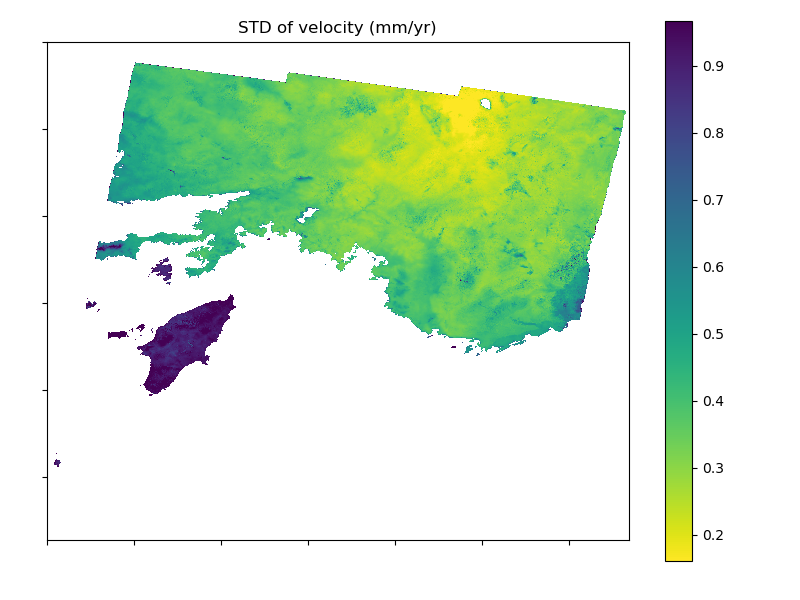

Supplement: Supplementary file 2 — Supporting Information S2 [file TECT-43-0-s001.zip › S3/138D_05325_TS/vstd.png]
